# Structural and Dynamic Differences between Calreticulin Mutants Associated with Essential Thrombocythemia

**DOI:** 10.3390/biom13030509

**Published:** 2023-03-10

**Authors:** Ragousandirane Radjasandirane, Alexandre G. de Brevern

**Affiliations:** Université Paris Cité and Université de la Réunion and Université des Antilles, INSERM, BIGR, DSIMB Bioinformatics Team, F-75014 Paris, France

**Keywords:** CALR mutations, molecular dynamics, essential thrombocythemia, chronic myeloproliferative neoplasms, blood cancer, disulfide bonds, calcium, Protein Blocks

## Abstract

Essential thrombocythemia (ET) is a blood cancer. ET is characterized by an overproduction of platelets that can lead to thrombosis formation. Platelet overproduction occurs in megakaryocytes through a signaling pathway that could involve JAK2, MPL, or CALR proteins. CALR mutations are associated with 25–30% of ET patients; CALR variants must be dimerized to induce ET. We classified these variants into five classes named A to E; classes A and B are the most frequent classes in patients with ET. The dynamic properties of these five classes using structural models of CALR’s C-domain were analyzed using molecular dynamics simulations. Classes A, B, and C are associated with frameshifts in the C-domain. Their dimers can be stable only if a disulfide bond is formed; otherwise, the two monomers repulse each other. Classes D and E cannot be stable as dimers due to the absence of disulfide bonds. Class E and wild-type CALR have similar dynamic properties. These results suggest that the disulfide bond newly formed in classes A, B, and C may be essential for the pathogenicity of these variants. They also underline that class E cannot be directly related to ET but corresponds to human polymorphisms.

## 1. Introduction

Essential thrombocythemia (ET) is one of the principal chronic myeloproliferative neoplasms (MPNs). It progresses when megakaryocytes in the bone marrow produce an excess number of platelets [1,2]. ET has an incidence of 1.2 to 3.0 per 100,000 population per year [3]. ET has chronic courses complicated by thrombohemorrhagic events, systemic symptoms, splenomegaly, and progression to myelofibrosis and/or blast phase [4,5,6,7,8,9]. Various drugs are prescribed to normalize the platelet count in patients at high risk of ET. However, the superiority of either interferon-α or hydroxyurea has not yet been established [10,11].

ET-specific driver mutations were recently discovered. The first localized protein is a famous tyrosine kinase enzyme, the Janus kinase 2 (JAK2) protein, and, more specifically, it is JAK2V617F [12]. It is also the major player in another MPN, polycythemia vera [13], characterized by excessive red blood cell levels. JAK2 is involved in the signal transduction of erythropoietin and thrombopoietin; JAK2V617F is represented in 60–65% of ET patients [14,15]. The second protein is a transmembrane receptor interacting with cytosolic JAK2, namely, the thrombopoietin receptor protein or MPL [16]. MPL is involved in 4 to 10% of cases [17]; its main variant is MPLW515K, which is located at the border of the membrane [18].

A third protein was characterized in 2013 [19,20], namely, calreticulin (CALR) [21,22,23,24]. CALR is an endoplasmic reticulum (ER) Ca^2+^-binding chaperone that functions in the folding and assembly of glycoproteins [25,26,27,28,29,30]. CALR binds half of the ER Ca^2+^ [31,32,33] and has specificity for monoglycosylated N-glycans on substrate proteins, which are transiently acquired during glycoprotein maturation in the ER. CALR contains three domains: (i) a lectin-like N-terminal domain (N-domain) containing glycan-binding and high-affinity calcium-binding sites [34], (ii) a central elongated hairpin-like P-domain containing a co-chaperone-binding site [27], and (iii) an acidic C-terminal domain (C-domain), which contains multiple low-affinity Ca^2+^-binding sites [32]. The three domains have been crystallized separately, and a cryo-EM structure has been proposed as a global model with the end of the C-domain missing and the beginning of the C-domain mainly composed of helical residues [35]. CALR mutations (CALRms) associated with ET always affect the ninth exon [36]. This region corresponds to the third and last structural domains of CALR (named the C-domain). CALRms consist of deletions and/or insertions; they often have a one-base-pair shift, leading to significant changes in the C-domain [37,38]. From a physicochemical point of view, CALRms become more basic. The C-domain also loses the ER retention motif KDEL; it does not stay in the ER but is exported outside the megakaryocyte and then interacts with MPL. This binding leads to platelet proliferation [7,39].

CALRm accounts for 25 to 30% of ET patients and ∼20% of MPN patients [40]. The two most common CALRms account for 80–85% of cases; they are c.1092_1143del, p.L367fs*46, designated as the classical type 1, and c.1154_1155insTTGTC, p.K385fs*47, designated as the classical type 2. Different studies have shown a correlation between the CALR mutation type and the prognosis value for the stratification of thrombotic risk in ET patients [41,42,43,44]. The complications originally described only for the JAK2V617F mutation are increasingly being described for CALRm patients, such as stroke [45] or splenic mass [46].

To have a better outlook on CALRm, we have proposed a specific database named CALR-ETdb (https://www.dsimb.inserm.fr/CALR-ET, accessed 20 February 2023) [47] that aggregates all CALRms from the COSMIC database [48] and bibliographic searches. Originally composed of 155 CALRms, it has now been updated to 169. CALR-ETdb is searchable and provides information not only in terms of references and types but also of structural properties with secondary structures and three-dimensional structural models.

Thanks to this large number of CALRms, we propose a new classification of CALRm sequences defining five different classes and some surprising underlying properties. Hence, class A aggregates 96% of the former type 1 (and those that are 1-like), while class B comprises only 64% of the former type 2 (and those that are 2-like), i.e., 1/3 of them belong to other classes. Class C is mainly composed of the former other type. The unexpected cases were (i) class D with 4% of type 1, 8% of type 2, and 5.6% of other and which is associated with a particularly short length, and (ii) class E with no type 1, 28% of type 2, and 3.7% of the other type. Both classes have only point mutations that do not induce any frame change, and for the last of these, even the ER retention signal is maintained.

With loss of the retention signal in the ER (KDEL), CALRms cannot remain. However, another essential point is that CALRms must be in dimeric form to bind to MPL at the membrane surface of megakaryocytes [49,50,51]. It has been shown that the homomultimerization of mutant calreticulin is an obligatory prerequisite for MPL binding and activation. Recently, Raghavan’s group investigated the relevance of disulfide-bond-mediated interactions in CALRm multimerization in primary patient platelets and human cell lines expressing recombinant mutant CALRms [52,53]. They showed that new cysteine residues in the C-terminal ends of CALRm mutants contributed to CALRm (type 1) and MPL interactions by forming disulfide bridges, both in primary patient platelets and also in human cell lines expressing recombinant CALRm mutants.

In the present research, we have extended this analysis to underline if the ability of these different classes to dimerize is found for our different CALRm classes. Indeed, different dynamic properties could be related to oncological impacts. For this purpose, molecular dynamics simulations were performed for the reference CALR and five CALRm classes. We tested the monomer and dimer for CALRm and for the wild-type CALR. The results clearly show that (i) the protocol was correct, (ii) the dynamics of classes A, B, and C are slightly different and are stable as dimers, and (iii) that classes D and E cannot be stable as dimers. These last two seem to not be associated directly with MPL binding. Therefore, they also may not be directly linked to ET and platelet proliferation.

## 2. Materials and Methods

### 2.1. Dataset

**Figure 1:**
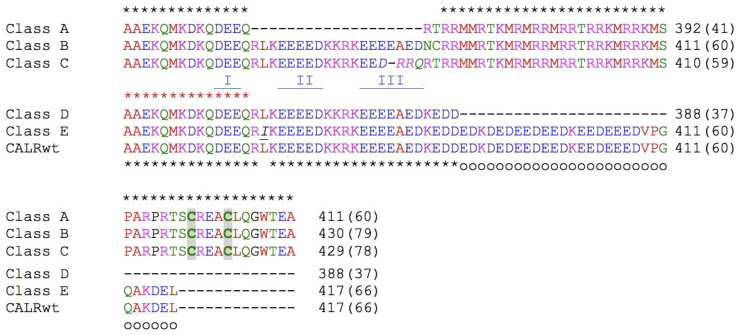
The 6 different CALR C-domains used in the study. The sequences of CALR wild-type (CALRwt) and the 5 CALRms (class A: p.L367Tfs*46, CALR-ETdb variant 20; B: p.K385Nfs*47, variant 10; C: p.E380Dfs*51, variant 5; D: p.E389*, variant 16; and E: p.L367I, variant 3) were aligned with CLUSTAL Omega (and modified manually) and are shown with red stars to indicate the positions always found, with black stars, if only found for one of the two sub-clusters, and with circles, if only found between class E and CALRwt. The residues that differ between these two are in italics and underlined. The positions of the three stretches are also shown (I: found in all classes, II: found only in classes B and C, and III: complete only in class B). CALR C-domain begins with alanine 352; length of each CALR is provided at the end of each line.

The CALR sequence was retrieved from UniProt (UniProt ID: P27797) [54]. For clarity, the wild-type CALR was named CALRwt. ET impacts the C-terminus of the CALR protein, i.e., the C-domain corresponding to the third (and last) domain; it is the ninth exon. The C-domain extends from alanine 352 to leucine 417 of CALRwt. The 5 other sequences of CALRms were taken from CALR-ETdb. Each one corresponds to one of the five classes described in [47], with class A being p.L367Tfs*46 (and corresponding to classical CALR type 1), CALR-ETdb variant 20; class B being p.K385Nfs*47 (and corresponding to classical CALR type 2), CALR-ETdb variant 10; class C being p.E380Dfs*51, CALR-ETdb variant 5; class D being p.E389*, CALR-ETdb variant 16; and class E being p.L367I, CALR-ETdb variant 3. Figure 1 summarizes all the different studied systems.

### 2.2. Structural Models

The structural templates to model CALRwt were selected thanks to PSI-BLAST software [55] used with the Protein Data Bank [56]. It makes it possible to detect proteins with sequences close to the query sequence and with structures that can be used for modeling, namely, structural modeling. The mining of the different databases was performed with PSI-BLAST with an e-value greater than 0.005.

**Figure 2:**
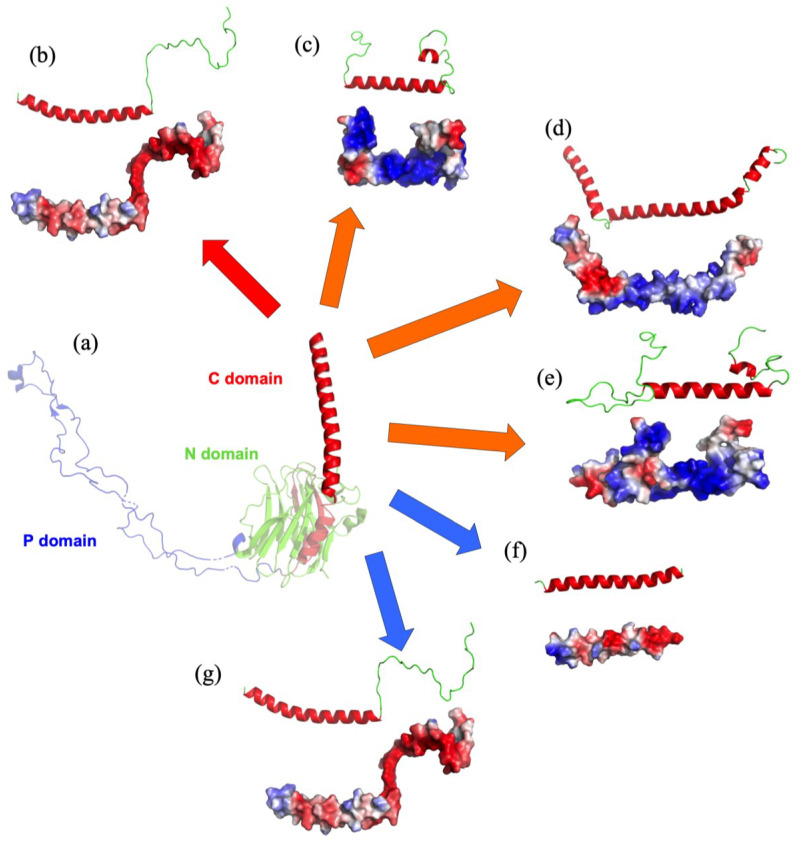
The 6 different structural models of CALR C-domains. (**a**) Calreticulin structure from PDB, ID 6ENY a chain E structure [35] with the three determined domains (N-, P-, and C-domains, the last of which is incomplete); and (**b**–**g**) provide the different modeled C-domains with (up) secondary structure and (down) electrostatics visualization: (**b**) CALRwt, (**c**) class A, (**d**) class B, (**e**) class C, (**f**) class D, and (**g**) class E.

CALRwt was based on the calreticulin partial structure (PDB ID 6ENY chain E structure [35] from the Protein Data Bank [56]) and completed (please notice that the C-domain is identical to the one from the PDB, ID 5LK5 [57]). The CALRm structural models were taken from our database CALR-ETdb (https://www.dsimb.inserm.fr/CALR-ET accessed on 17 December 2022) [47]. The modeling of the 3D models was performed with MODELLER v.10.0 [58]. The selection of the best structural model was mainly based on the renowned DOPE approach [59]. The three-dimensional structure and structural models were visualized using PyMOL (the PyMOL Molecular Graphics System, Version 1.7.2.2 Schrödinger, LLC) [60]. Other modeling approaches were also used, i.e., I-TASSER [61], Robetta [62], AlphaFold2 [63], and Phyre2 [64], but without satisfying results compared with a human-supervised approach using MODELLER (see the Discussion section). Figure 2 shows all the modeled systems (they are all provided in Data S1).

### 2.3. Dimer Structural Models

Following the same principle, dimers of every system were built. For classes A, B, and C, CALRms with two novel cysteines appeared in the sequences due to the frameshift. They have been shown to have a determinant role in CALR dimerization and MPL receptor activation through the CALRm dimers [52]. Thus, the dimeric form of CALRms is based on two disulfide bonds between chains using the two cysteines within the CREAC motif at the end of the sequence. MODELLER [58] was used with two monomer chains and a specific constraint between cysteines to create the disulfide bridges. For CALRwt, and classes D and E, their sequences have no cysteines to form disulfide bonds. The dimers were built using distance constraints to keep each chain close enough together to be comparable with other CALRm dimers with disulfide bonds. For all these models, the best DOPE score [59] was used to select the best model (they are all provided in Data S1). The two chains will be named chain 1 and chain 2, respectively, in the text, as they often did not share the same dynamics.

### 2.4. Molecular Dynamics

Molecular Dynamic (MD) simulations were performed using GROMACS 2020.4 software [65] with the CHARMM-36 forcefield [66]. Before starting any simulation experiments, each structural variant was energy-minimized for 500 steps of the steepest descent and 500 steps of the conjugate gradient optimized with the SHAKE algorithm, using the GROMACS suite. CALRwt and 5 different CALRms, both in monomeric and dimeric forms, were soaked in a rhombic dodecahedral simulation box with TIP3P water molecules. A total of 13 calcium ions (Ca^2+^) were added as positive counter-ions into the system, in order to neutralize it, for CALRwt and CALRm class E because these two systems are negatively charged. The other variants (classes A, B, and C) had chloride ions (Cl^-^) as counter-ions because they are positively charged.

The MD protocol was standardized through our previous works [67,68]. After 1 nanosecond (nsec) of equilibration (with position restraints on the proteins), each system was simulated through multiple classical independent production runs ranging from a total of 100 to 500 nanoseconds, as in [67]. The equilibration consists of one step with an NVT system and three more with an NPT system (with a gradual decrease in position constraints). During the first step in an NVT system and the second step in an NPT system, the protein is totally constrained and cannot move, and the equilibration affects the water molecules. In the third and fourth steps in an NPT system, the constraints on the protein are slowly released. Molecular conformations were saved every 100 picoseconds for downstream analysis. The first 5 nsec of each MD simulation was discarded as the residues at the extremities could not be taken into calculations. Trajectory analyses were carried out with GROMACS software and in-house Python and R scripts. Root-mean-square deviations (RMSDs) and root-mean-square fluctuations (RMSFs) were calculated for Cα atoms only.

### 2.5. Molecular Dynamics Analysis

The analysis of MD was carried out using classic tools, for example, the RMSD and RMSF, and other more innovative ones such as PBxplore [69], a tool developed within the team. PBxplore makes it possible to analyze protein blocks throughout the MD. MD analysis [70], a Python library, was used to compute distance calculations between Ca^2+^ ions and their binding sites during the simulations.

The RMSD (root-mean-square deviation) makes it possible to see how a structure varies during the dynamics by comparing it with the starting structure (reference). For each frame, an average of the differences between the reference positions and the positions of the current frame is performed in order to obtain an RMSD value per unit of time.

The RMSF (root-mean-square fluctuation) is similar to the RMSD. It makes it possible to determine the fluctuation of each residue following the same principle as for the RMSD, i.e., a comparison with a reference that is the average position of each residue and, thus, measure the difference between the current position and the average position in order to have a flexibility value per position.

Protein Blocks (PBs) are a structural alphabet composed of 16 local prototypes [71]. Each specific PB is characterized by the φ, ψ dihedral angles of five consecutive residues, with each PB assignment focused on the central residue. Obtained through an unsupervised training approach and performed on a representative non-redundant databank, PBs give a reasonable approximation of all local protein 3D structures [72]. PBs are very efficient in tasks such as protein superimpositions [73] and MD analyses [74]. They are labeled from *a* to *p*: PBs *m* and *d* can be roughly described as prototypes for an α-helix and central β-strand, respectively; PBs *a* to *c* primarily represent β-strand N-caps; PBs *e* and *f* represent β-strand C-caps; and PBs *a* to *j* are specific to coils, PBs *k* and *l* to α-helix N-caps, while PBs *n* to *p* to α-helix C-caps. Each PB assignment was carried out using our PBxplore tool (available at GitHub) [69].

PB assignments were undertaken for each residue of the C-domain and over every snapshot extracted from the MD simulations. The equivalent number of PBs (*N_eq_*) is a statistical measurement similar to entropy that represents the average number of PBs for a residue at a given position. *N_eq_* is calculated as follows [71]:

$$N_{eq}=exp\left(-\overset{16}{\underset{x}{\sum }}f_{x}\;ln\;f_{x}\;\right)$$

where *f_x_* is the probability of PB *x*. An *N_eq_* value of 1 indicates that only one type of PB is observed, while a value of 16 is equivalent to a random distribution. To underline the main differences between one system and another one for each position, the absolute difference Δ*N_eq_* between corresponding *N_eqs_* values was computed.

However, since the same Δ*N_eq_* value can be obtained with different types of blocks in similar proportions, we defined a complementary measure, ΔPB, that evaluates a change in the PB profile by calculating the absolute sum of the differences for each PB between the probabilities of a PB *x* to be present in the first and the second forms (*x* goes from PB *a* to PB *p*). *ΔPB* is calculated as follows [67]:

(1) $$∆PB=\overset{16}{\underset{x=1}{\sum }}\left|f_{x}^{S1}-f_{x}^{S2}\right|$$

where *f^S1^_x_* and *f^S2^_x_* are the percentages of occurrence of a PB *x* in the first (S1) and the second system (S2), respectively. A value of 0 indicates a perfect PB identity between the 1st and 2nd systems, while a score of 2 indicates a total difference.

It should be noted that all the protocols (modeling, molecular dynamics, and analyses) were similar for all the systems, for both monomers and dimers.

## 3. Results

### 3.1. Analysis of CALRwt Monomer Dynamics

CALRwt is a calcium-binding chaperone residing in the endoplasmic reticulum (ER), thanks to the KDEL motif localized at the end of its C-terminus, which functions as an endoplasmic reticulum retention signal [75]. As the CALR C-domain is incomplete in the PDB structure (PDB ID 6ENY chain E structure [35] (see Figure 2A)), it was previously completed with comparative modeling, with MODELLER [58] and PSIPRED [76] predictions used as constraints (see Materials and Methods section and Figure 2B). The electrostatics shows highly negative values in the C-terminus region.

This first part consists of using the wild-type reference form of CALR to assess whether it is possible via MD to reproduce the physiological behavior of the C-domain of CALR, i.e., to capture Ca^2+^ ions. We therefore simulated the dynamics of CALRwt in monomeric form over 500 nsec. Thirteen Ca^2+^ ions were added to neutralize the negatively charged system due to numerous aspartic acid and glutamic acid residues.

**Figure 3:**
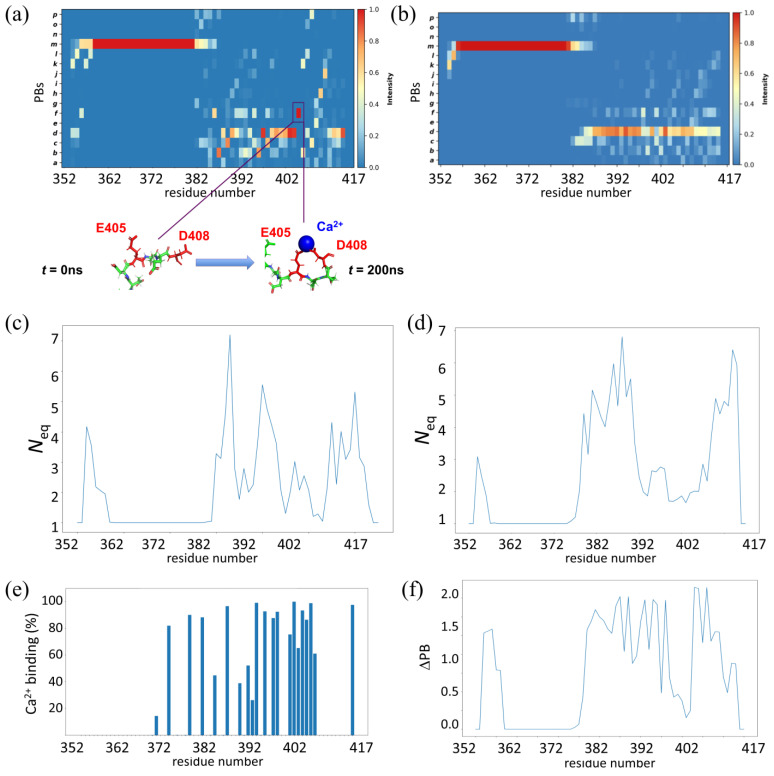
MD of CALRwt. (**a**) PB analysis of CALRwt with Ca^2+^, with a focus on the Ca^2+^ bound by residues E405 and D408; (**b**) PB analysis of CALRwt with Na^+^; Neq of CALRwt (**c**) with Ca^2+^ and (**d**) with Na^+^; (**e**) site occupation of Ca^2+^ (for CALRwt with Ca^2+^); and (**f**) ΔPB between MD with Ca^2+^ and with Na^+^.

The RMSD values reached a plateau around the correct value of 1.25 Å from 20 nsec to the end of the simulation (these same results were found for other replicates, but the data are not shown), i.e., the simulation stabilized quickly and could therefore be used. The MD simulations were analyzed in light of the RMSFs and Protein Blocks (PBs). For the map of the PB highlights regions with high flexibility and rigid regions, see Figure 3a,c. In particular, the beginning (residues 352 to 360) and end (residues 382 to 386) of the helix show a high variation in PBs with *N*_eq_ values of four and seven, respectively (see Figure 3c), which indicates that the start of the helix tends to be a deformable region, whereas the end of the helix appears to be disordered (as defined in [77]). The helical region is characterized by PB *m* (see Figure 3a). The second part of the structure is constituted of unstructured regions (residues 387 to 417), but some residues show very high intensities in terms of PB. For instance, residue E405 fixes calcium ions with another residue (D408), which stabilizes its local conformation (see Figure 3a).

Initially, CALRwt was without any Ca^2+^ ions fixed onto it. However, after 50 nsec, almost all Ca^2+^ ions were bonded to the CALRwt structure (see Videos S1 and S2). This produces local structural constraints from calcium ions bonded between two residues (see Figure 3e). Hence, the region between residue 392 and residue 405 has a high density of calcium fixation within a radius of 3 Å from each residue. This density is also visible in the PB map with a high intensity of blocks (in red-orange) (see Figure 3a) as well as in the RMSF and *N*_eq_ plot with a decreasing value in this region (see Figure 3c). The distribution of the binding sites for Ca^2+^ ions does not seem to be deterministic. Indeed, the binding sites between the simulations of 400 nsec and 100 nsec (see Figure S1a,b) are different, which led to different PB maps; it also led to different local conformations (see Figure 3a,c and Figure S2a,b).

To try and check the impact of calcium ions on the structure, we simulated the same system in similar conditions but with 26 Na^+^ ions instead of 13 Ca^2+^ ions. Na^+^ ions do not bind to the structure of CALRwt, which leaves the CALRwt structure without any structural constraints. As with the simulation with Ca^2+^ ions, the end of the helix was also highly flexible with an *N*_eq_ value between six and seven (see Figure 3d). The second part of the structure could not be stabilized with Ca^2+^ ion binding; however, the PB profile for the unstructured regions remained around PB *d* (see Figure 3b) and related conformations, with some high *N*_eq_ values often higher than four. With only Na^+^, replicates of the simulation provided similar profiles (see Figure 3b,d and Figure S2c,d and Video S3).

Local structural differences between the two systems (with Ca^2+^ or with Na^+^) can be compared using Protein Blocks. Ca^2+^ binding induces local conformations that are not present in the simulation of CALRwt with Na^+^ ions. Indeed, the region around residue 407 has a ΔPB superior to one (see Figure 3f), indicating that these simulations have different PB distributions for this region. In fact, a Ca^2+^ ion bound to residue E403 and L417 and provokes a specific fold that is strongly different from the CALRwt Na^+^ simulation (see Figure S3). Some regions are still found to be similar between the two systems. The region around residue 401 has a low ΔPB, i.e., this region is less constrained due to calcium ion binding. An additional half-microsecond simulation was conducted with Ca^2+^, and, in addition to Na^+^ and Cl^-^, the CALRwt behaviors followed those of the simulations with only Ca^2+^ (see Figure S4) as these ions bind very quickly, as seen previously.

The results of this first part show that the CALRwt models allow a good fixation of Ca^2+^ ions. Therefore, these simulations properly mimic the natural role of CALRwt in binding Ca^2+^ ions. These MD simulations also underline the strong dynamic differences between a calcium medium and a sodium medium. For the first, the interactions strongly stabilize local conformations, while the second makes it possible to leave the disordered tendencies of the C-domain C-terminus. These data suggest that this approach can be applied to different CALRs.

### 3.2. Frameshifts Induce Distinct Dynamics for CALRm Classes A, B, and C

The CALR variants were classified into five classes from A to E (see Figure 1). Classes A, B, and C are characterized by frameshifts and loss of the endoplasmic reticulum retention signal. They are characterized by a strong change in their charges (see Figure 2C–E), and they are not supposed to be able to bind Ca^2+^ ions anymore, having become very positive. They also carry new cysteines to allow their dimerization [52]. The last two classes do not share these cysteines. The class E sequence is similar to CALRwt, with one or more punctual mutations (while keeping the ER retention signal), and class D is roughly half of CALRwt (without the ER retention signal).

**Figure 4:**
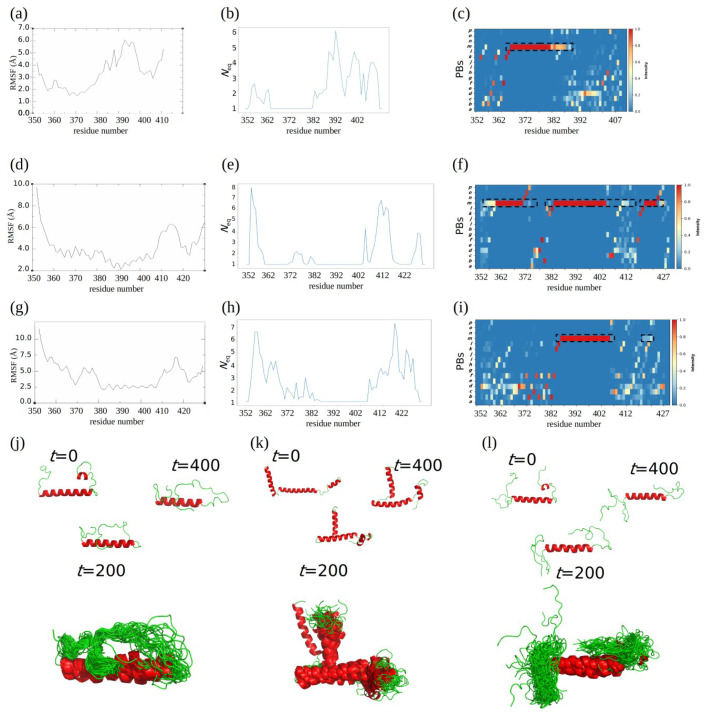
MD of CALRm classes A, B, and C. (**a**,**d**,**g**) Show RMSF values; (**b**,**e**,**h**) show *N*_eq_ values; (**c**,**f**,**i**) PB analysis of (**a**–**c**) class A, (**d**-**f**) class B, and (**g**–**i**) class C; (**j**–**l**) show visualizations for 3 times (0 nsec, 200 nsec, and 400 nsec) of MD simulations (up) and superimposition of multiple observed conformations (down) of (**j**) class A, (**k**) class B, and (**l**) class C. Please note that the choice of snapshots after that of the initial conformation is arbitrary but allows us to note the movements of the systems well (see also the Supplementary Videos).

In this second part, we focus on the first three CALRm classes as monomers. They correspond perfectly to what is expected from CALRms associated with ET. The models were built from the available CALRwt structure and completed as before, with secondary structural constraints taken from PSIPRED. The selected models, therefore, have a perfect match with the predicted helical residue and the predicted loops. The simulations were carried out in a similar way as with CALRwt. The only difference was the charges being different to neutralize them, with Cl- ions having been added. The example of CALRm class A is the classic canonical type I [19,20]. CALRm class A (see Figure 4a–c,j and Video S3) is characterized by a unique main α-helix ranging from residue 366 to 388 (see Figure 4c). The beginning of the fragment is quite rigid with a succession of small turns characterized by PBs *k*, *b*, and *f* and low values of less than 2.5 (see Figure 4b). The helix is highly rigid with an *N*_eq_ value equal to one, beginning with the N-cap PBs *fkl* (positions 362 to 364) to position 382, the C-cap (positions 383 to 388 are less constrained, with *N*_eq_ values of two). Positions 395 and 402–404 are the only positions with some local constraints (*N*_eq_ values of two or less), while the rest of the C-termini region is highly flexible, with some *N*_eq_ values of higher than four (see Figure 4b). The RMSF values are well correlated with this analysis. The rigid helix is associated with the lowest RMSF values (see Figure 4a), while the C-termini has the highest RMSF values. This behavior is well described in Figure 4j, which shows that since the initial model (n = 0 nsec), the adjacent parts move, especially the C-terminal part (n = 200 and n = 400 nsec). The superposition of a large number of conformations shows the particularly flexible aspect of these regions around the helix of class A well.

Thus, CALRm class A is characterized by a single rigid helix of about 20 residues, the end of the mutated C-domain being particularly flexible with few local constraints.

The example of CALRm class B corresponds to the classic canonical type II [19,20]. Very surprisingly, the dynamics of the CALRm class B monomer is strongly different from that of class A. CALRm class B has three helices (see Figure 4d–f,k and Video S5); the first helix spans from residue 354 to 374, the second and larger helix from residue 379 to 414, and the last and smaller one from residue 417 to 425 (see Figure 4d). The N-cap of CALRm class B is disordered (with an *N*_eq_ value of eight at position 354), the N-cap of the first helix (positions before 358) is unstable, while the rest of this helix is rigid (with *N*_eq_ values of one), and even the transition of the second helix is highly constrained with PB series *nopadcdfblkl* (positions 372 to 382 with *N*_eq_ values of less than two). The second long helix is rigid till position 401. A disordered region is then found between positions 406 and 417. The last helix is well-defined with an *N*_eq_ of one. Due to the disordered regions, the RMSF values are higher and could provide a false impression of instability. This is well explained in Figure 4k, which shows the separated movements of the different helices that are maintained as coherent.

Thus, CALRm class B is characterized by 3 rigid helices of about 14, 22, and 6 residues, respectively, and with one main disordered region of 10 residues.

The example of CALRm class C came from the former other type [19,20]. It has the length of the CALRm class B example but looks like CALRm class A, with only one long helix ranging from residues 384 to 406 (see Video S6). The original second smaller one (positions 418 to 422) disappeared at the beginning of the simulations. The class C dynamics is highly particular with highly flexible/disordered N- and C-termini with *N*_eq_ values of higher than six, associated with the high sampling of different local conformations. Before the α-helix, beginning at position 369, local constraints become important with the series of PBs *dddfbdcfbfbdfbf*. This series is rarely observed in ordered proteins, but here, is found with a low *N*_eq_ (of less than two). As for the CALRm class B example, the RMSF values can be confusing as disordered regions can provide the wrong impression of very high values. This is, again, well presented in Figure 4k. The three different times of the simulation show the flexibility of the regions outside the helix (and the constraints observed before it) and the superimposition of the complexity of these different zones well.

Thus, CALRm class C is characterized by a single rigid helix of about 19 residues, with 10 residues before it having constrained local conformations and the end of the mutated C-domain being highly flexible.

The results of this second part show that the CALRm models of classes A, B, and C exhibit unfolding at the ends of the α-helices, with the presence of extremely flexible zones (see Videos S4–S6). The reductions in the α-helix size are identical to those observed for CALRwt dynamics with Na^+^ ions (this is not observed with Ca^2+^ ions (see Video S1)). The second point of interest is the difference in behavior between the three classes. They are clearly distinct in terms of dynamics. Class A (herein, represented by canonical type I) has an extremity that is highly flexible (*N*_eq_ > 4–6) with some more constrained residues (with *N*_eq_ values of two). Class B (herein, canonical type II) has three helices that decrease in size but remain present. Only one true region is disordered between the second and the last helix. We, therefore, have a set of three rigid bodies quite distinct from class A. Even more surprisingly, class C is composed, from the N- to C-termini, of (i) a disordered zone, (ii) a succession of local constraints that correspond to β-turns, followed by (iii) a central helix (corresponding to the second helix of class B), and then (iv) a very disordered C-terminus region (*N*_eq_ > 4–6). These three different behaviors were not too expected but are clear. It should be noted that Ca^2+^, Na^+^, and Cl^-^ mixtures were made without this changing the results of the MD.

### 3.3. Unexpected Similarities between CALRm Classes D and E and CALRwt Dynamics

This third part focuses on classes D and E. The former corresponds to a short form of the C-terminal of CALRwt, while the latter has only a point mutation with CALRwt. It was carried out using SCWRL software [78]. They are, therefore, very close to CALRwt, i.e., they are cysteine-free and therefore cannot be dimerized by the covalent bond of cystine. Ca^2+^ ions were, therefore, added, as with CALRwt, and all the parameters were identical to the previous simulations.

**Figure 5:**
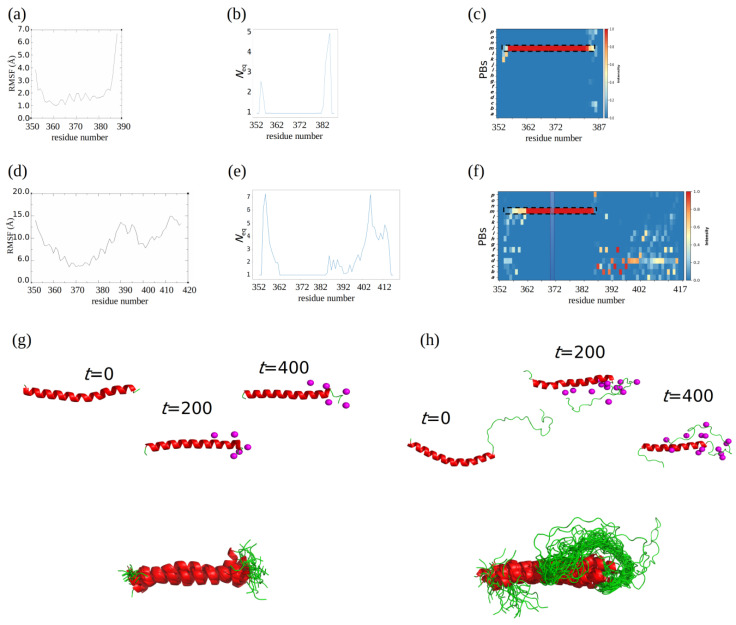
MD of CALRm classes D and E. (**a**) and (**d**) show RMSF values; (**b**) and (**e**) show *N*_eq_ values; (**c**,**f**) PB analysis of (**a**–**c**) class D and (**d**–**f**) class E; (**g**,**h**) show visualizations of 3 times (0 nsec, 200 nsec, and 400 nsec) of MD simulations (up) and superimposition of multiple observed conformations (down) of (**g**) class D and (**h**) class E.

CALRm class D is extremely short (see Figure 5 and Video S7). It is mainly composed of one long α-helix ranging from residue 353 to 386 (see Figure 5c). This helix remains stable during 400 nsec (with *N*_eq_ values of one), but its extremities are flexible with an *N*_eq_ value of around five for the end of the helix (see Figure 5b). The RMSF values reflect the same observation (see Figure 5a). Interestingly, some replicates show more flexibility in the N-cap than the C-cap region. Figure 5g shows how Ca^2+^ interacts with the C-cap region, and the superimposition underlines the great stability of CALRm class D.

The CALRm class E sequence is the same as that of CALRwt, with one residue substituted at position 367 (L367I). CALRm class E has one main α-helix from residue 352 to residue 386 (see Figure 5f and Video S8). As with CALRwt, it binds very quickly to Ca^2+^ ions (see Figure S5a,b). Surprisingly, the beginning of the α-helix is highly flexible and tends to be a disordered region with an *N*_eq_ value of seven (see Figure 5e). On the contrary, the end of the helix is quite stable with an *N*_eq_ value of two. This is found in all replicates. These observations do not correspond to the CALRwt dynamics with Ca^2+^ ions, wherein the N-cap was stable but not the C-cap of the α-helix (see ΔPB in Figure S5c). This unstructured conformation can be induced by the position of the mutation L367I (symbolized by a bar in the PB map in Figure 5f), having a potential allosteric effect on the stability of the helix. Ca^2+^ ions also bind to the structure of the class E variant, as with CALRwt, stabilizing the regions in the second part of the structure (indicated by the red blocks in the PB maps). However, in every case, the core of the helix is always highly rigid. We also notice that the Ca^2+^ site, as seen previously, often fluctuates, providing different local conformations (see Figure S5c). Figure 5h shows the complexity of the N-termini region conformations, even with Ca^2+^ ions.

Both classes are different from CALRm classes A, B, and C since they do not share any cysteines to form dimers through disulfide bonds. Moreover, the dynamics of class D does not look like the dynamics of the main classes. Indeed, the destructuring of the helix observed in class D is not on the same level as that for CALRm class A, B, or C but is closer to CALRwt behaviors.

CALRm class E has some differences from CALRwt, particularly in the stability of the helix and the beginning of the helix, which tends to be disordered. Nevertheless, class E shares some characteristics with CALRwt, such as calcium ions fixation, which stabilizes the structure in some regions, or the absence of cysteines in the sequence. Finally, CALRm class E is the only one that still possesses the KDEL motif. Hence, variants from class E would probably be maintained in the ER, as with CALRwt, without the capacity to induce ET by binding onto the MPL of megakaryocytes. We will look at this question in the following sections.

The results of this third part show that the CALRm models of classes D and E are (i) quite distinct from the dynamics of classes A, B, and C, and (ii) close to the dynamics of CALRwt. Like the latter, they interact with Ca^2+^ ions and therefore have similar local constraints.

### 3.4. CALRwt Dimer Does Not Stay as a Dimer

**Figure 6:**
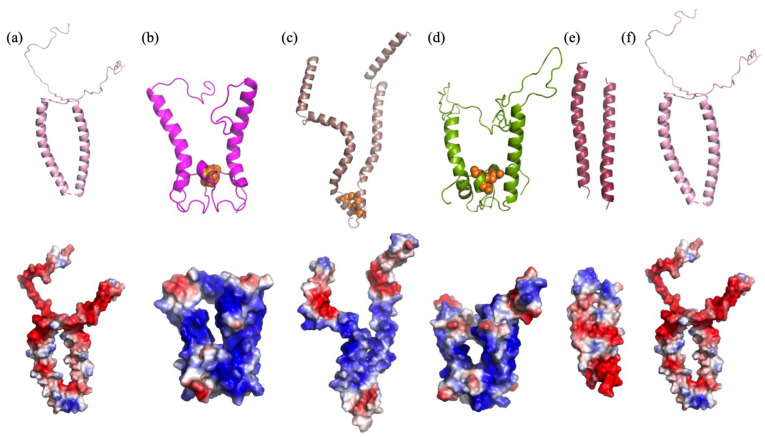
All the initial dimer systems. (**a**) CALRwt, (**b**) CALRm A, (**c**) CALRm B, (**d**) CALRm C, (**e**) CALRm D, and (**f**) CALRm E; (up) secondary structure visualizations and (down) electrostatics. (**b**–**d**) Cysteines implicated in disulfide bridges are indicated by orange balls.

In this fourth part, we focus on CALRwt as a dimer. As noted in the Materials and Method section, different options could have been considered to build the CALR dimers, such as docking. We preferred a constrained approach that is perfectly appropriate for the case of CALRms with disulfide bridges, i.e., CALRms A, B, and C (see Figure S6). We, therefore, used MODELLER for creating the disulfide bridges. The selected 3D models that were used for molecular dynamics were used as structural templates for both chains. We applied the same approach, with a distance criterion in the area corresponding to the cysteines, for CALRwt, and also for CALRm classes D and E, to start from comparable conformations (see Section 3.6). Figure 6 shows the results of the approach. It slightly modifies some of the 3D structural models, such as CALRm A (see Figure 2c and Figure 6b), but not significantly. The visualization of the electrostatics already shows that the charges are important and quite often opposed and at a short distance between the two chains.

**Figure 7:**
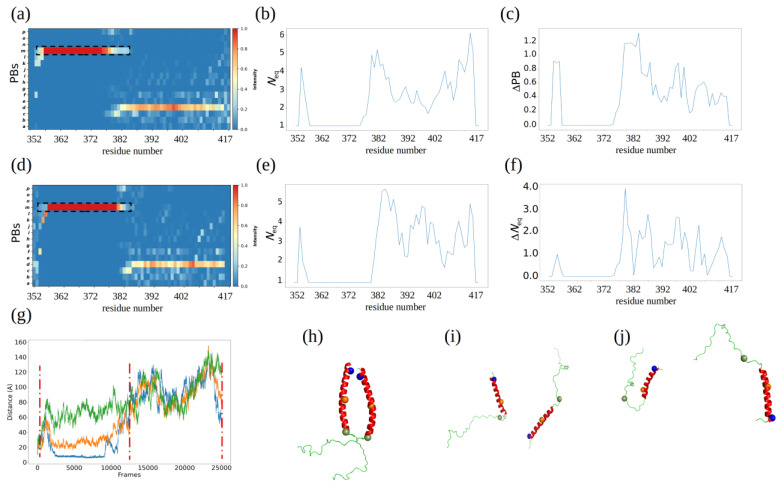
MD of CALRwt dimer. PB analysis of (**a**) chain 1 and (**d**) chain 2; (**b**) and (**e**) show corresponding *N*_eq_; (**c**) ΔPB and (**f**) Δ*N*_eq_ between the two chains; (**g**) distances between different positions of the two chains (in blue, residue 356, in orange, 371, and in green, 386); and (**h**–**j**) 3D conformations observed during the simulations (positions in the simulation shown with red bars in (**g**)).

The dynamics of the dimeric form of CALRwt was simulated for 500 nsec (with 2 replicates of 250 nsec (see Figure 7)), using Ca^2+^ ions to neutralize the system. Each chain has stable dynamics thanks to Ca^2+^ ions binding to the structures. Indeed, the helix remains mainly well structured. The Ca^2+^ ions binding to the structure allows interaction between chains through Ca^2+^ ions, similar to the interaction seen between residues on the monomeric form of CALRwt. A simulation of the CALRwt dimer was computed using Na^+^ ions instead of Ca^2+^ ions over 250 nsec. The dynamics of each chain is quite close to the dynamics of the CALRwt monomer with Na^+^ ions (see Figure S2c and Figure 7a,d). Indeed, the ΔPB values are below one for almost the whole structure, meaning that they share similar PBs between the chains and the monomeric form of CALRwt with Na^+^ ions (see Figure S7a–d). This also indicates that the instability of the C-cap helix of both chains is at the same level as that observed with the monomer (see Figure 3b,e and Figure 7b,e).

As in the previous systems, both chains have different dynamics (see Figure 7c,f) sampling different local conformations and flexibility levels. The distance computation shows a clear separation process for the dimer of CALRwt after 100 nsec (see Figure 7g). The two chains are not able to stay together, as seen in the evolution of the two chains (see Figure 7h–j). These results clearly show that the C-domain of CALRwt cannot simply dimerize and that its natural form must be monomeric.

### 3.5. Dimerization of CALRm Classes A, B, and C Mediated by Disulfide Bonds

In this fifth part, we focus on the first three CALRm classes as dimers. The dimers were created with MODELLER (i) using previous modeled classes and (ii) adding specific disulfide constraints between cysteines. These systems correspond to what is expected in CALRms associated with ET [52,53]. The simulations were carried out in a similar way as with CALRm classes A, B, and C with an appropriate number of Cl^-^ ions.

The dynamics of the CALRm class A dimer was simulated over 500 nsec to analyze the structural impact of having disulfide bonds. The dynamics of each chain is distinct with some local similarities (see Video S9).

**Figure 8:**
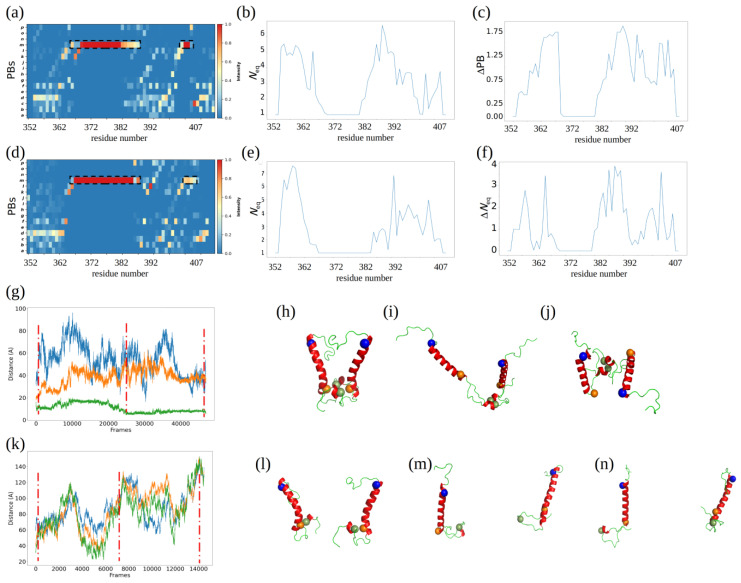
MD of CALRm dimer class A. (**a**–**j**) CALRm dimer class A with disulfide bridge, and (**k**) to (**n**) without disulfide bridge. PB analysis of (**a**) chain 1 and (**d**) chain 2; (**b**,**e**) show corresponding *N*_eq_; (**c**) ΔPB and (**f**) Δ*N*_eq_ between the two chains; (**g**,**k**) distance between different positions of the two chains (in blue, residue 366, in orange, 385, and in green, 400); and (**h**–**j**) and (**l**–**n**) show 3D conformations observed during the simulations (positions in the simulation shown with red bars in (**g**,**k**), respectively).

The main helix of chain 1 (see Figure 8a) appears to be more unstructured than that of chain 2 (see Figure 8d), especially at the beginning (residues 365 to 370) and the end (residues 382 to 387) of the helix, with *N*_eq_ values of one (see Figure 8b). The second helix remains mainly structured for chain 1, with *N*_eq_ values of one. On the contrary, the main helix of chain 2 is well-structured, whereas the second helix has a more variable PB profile and tends to be flexible, with an *N*_eq_ value of around four. The ΔPB values in these regions are superior to one, which means that they have different PB distributions (see Figure 8c). Despite these structural differences between the chains, they remain steadily linked through disulfide bonds, which maintain the dimeric form of CALRm class A. This linkage is probably responsible for the stabilization of the second helix of each chain since they remain mainly structured, unlike the dynamics of class A in monomeric form. Indeed, a comparison with the previous monomer of CALRm class A showed that the flexibility is distinct, with Δ*N*_eq_ values often between two and six, underlining a difference in terms of flexibility behaviors with monomeric forms (see Figure S8b,d). In a similar way, outside the helical regions, ΔPB often reaches values higher than 1.5, underlining that distinct conformations are explored due to the dimerization of the chains.

Indeed, this dimer is highly dynamic as it does not seem to like to stay as a dimer. A distance calculation between the two chains was performed to observe changes in the distance between the two chains during the simulation. Three positions were selected—residue 366 on chain 1 (the blue color sphere), residue 385 (the orange color sphere), and residue 400 (the green color sphere)—which correspond to the cysteines implicated in the disulfide bridge. The distance between the N-terminal of each chain varies between 25 and 85 Å (blue sphere). The distance in the second position varies between 20 and 50 Å (orange sphere), while the distance in the disulfide bond position remains always very high for a cystine, i.e., around 7 Å. It would have been graded as a bad-grade cystine [79,80]. In fact, the variation in the distance diminishes as we go from the N-terminal to the C-terminal sequence where the disulfide bonds are located. Indeed, positive electrostatic patches from the middle of both chains (see Figure 6b) induce a repulsive force, which can explain the relatively high distance between them before reaching the disulfide bonds. These observations suggest that disulfide bonds are required/essential to maintain the dimer form; otherwise, the electrostatic forces between chains would have made it impossible, and both chains would have been separated.

To ensure this result, a new system was made by reducing the disulfide bridge to have free cysteines. A short molecular dynamics of 140 nsec was performed and gave very clear results. Again, the helix retains its coherence quite well, and the rest strongly samples the conformational space quite significantly (see Figure S9 and Video S10) but corresponding to what was seen with the monomer. On the other hand, the dimer does not hold at all. From the first few seconds, the two chains repel each other, as suggested in the previous paragraphs. The distance variations range easily from 20 A to over 100 A, as seen in Figure 8k. The visualizations (see Figure 8l–n) show that they have become fully independent again and that the disulfide bridge is essential to maintain the dimerization of the C-domain of CALRm class A.

**Figure 9:**
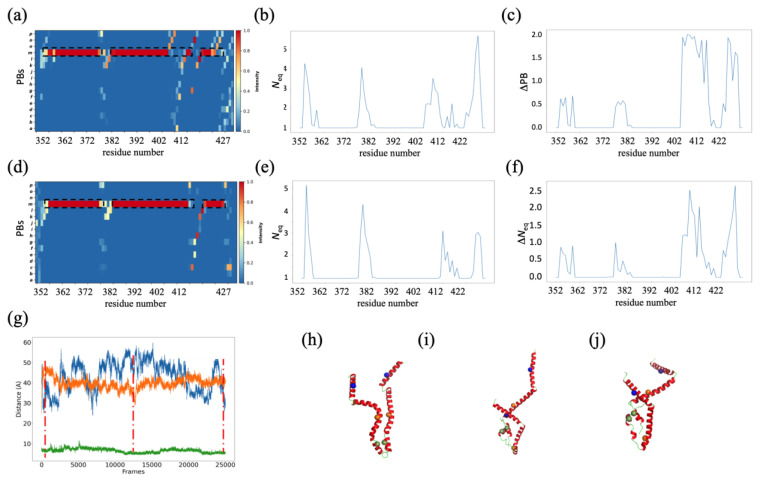
MD of CALRm dimer class B. PB analysis of (**a**) chain 1 and (**d**) chain 2; (**b**) and (**e**) show corresponding *N*_eq_; (**c**) ΔPB and (**f**) Δ*N*_eq_ between the two chains; (**g**) distance between different positions of the two chains (in blue, residue 366, in orange, 400, and in green, 419, the last being the cysteine implicated in the disulfide bridge); and (**h**–**j**) 3D conformations observed during the simulations (positions in the simulation shown with red bars in (**g**)).

The dimeric form of CALRm class B was modeled in the same way as the dimeric form of class A, its dynamics simulated over 500 nsec (2 times 250 nsec, as seen in Videos S11 and S12). Both chains have some differences in the PB distribution, especially on the main helix (see Figure 9a,d). Indeed, chain 1 has the main helix C-cap unstructured (from residue 406 to 410). The two other helices remain mainly structured. The helices in chain 2 are highly stable, *N*_eq_ plots underline mainly the loop regions (see Figure 9b,e), The Δ*N*_eq_ is more limited than that for the previous system as the helix limits local fluctuations (see Figure 8f and Figure 9f). The highest ΔPB value remains on the main helix C-cap and the third helix (see Figure 9c). As with the class A dimer, the disulfide bonds are likely stabilizing the helices around them, which allows these helices to be well-structured, unlike the dynamics of class B in monomeric form. Indeed, a comparison with the monomer shows that the intermediate regions have different flexibilities (see Figure S10b,d) and different sampled local conformations (see Figure S10a,c).

Similar to the class A dimer, distances were computed on three different anchors. The dimer quickly shows great movements that are translated into the subsequent separation of the two chains. The central position (orange ball) also moves quickly around 45 Å. It continues to oscillate during all the dynamics. The N-termini (blue ball) also starts to move away quickly at great distances (see Figure 9g–j) and oscillates between 40 and 60 Å. The main difference from the previous system is that the helices are numerous and move like small rigid bodies. After moving away, they do not move back together as their charges are identical and therefore unfavorable for interactions. Without the disulfide bridge, the two chains could not interact. A specificity of class B is the persistence of negative charges associated with the first helix that interact weakly with the positive charges of the second helix; this behavior is not observed in other cases.

Interestingly, the second MD replica shows exactly the same conformation between chains. However, the main and the third helices of chain 2 merge together to form only one helix that spans from residue 461 to 505. This surprising observation could perhaps highlight the long-range impact of disulfide bonds on stabilizing the structure.

The dynamics of the dimeric form of CALRm class C shows a high level of de-structuring in the first helix at the extremities and a large destruction of the second helix for both chains (see Video S13). The last observation follows the dynamics of CALRm class C in monomeric form. As with the other system, the dimeric form is not symmetric. Chain 2 shows more stable residues in helices in regard to the monomeric simulation. The distance between chains is quite low in the position close to the disulfide bonds and high in the N-terminal part of each chain.

The results of this fifth part underline that due to their charges, the CALRm classes A, B, and C cannot stay as dimers without the disulfide bridges. When the disulfide bridge is broken (see Video S10), the two chains separate instantly. Interestingly, this dimerization also influenced the dynamics of the protein chains. As is often seen in dimers, both chains are not equivalent (see Figure 6c, Figure 8c, and Figure 9c, with a high ΔPB between chains). On average, we can consider that there is even a slight stiffening of the systems (the *N*_eq_ remains quite high but slightly less than that for the monomer forms).

These results clearly show that the C-domain of these CALRms must dimerize simply with disulfide bridges and that otherwise, they must have monomeric behavior. Their sequences make them clearly distinct from CALRwt.

### 3.6. Incapacity to Dimerize of CALRm Classes D and E

This last part focuses on the CALRs that cannot dimerize through disulfide bonds, since they do not have any cysteines in their sequences. This includes CALRm classes D and E. As previously, the models of the monomers were used for the dimers. However, due to the absence of cysteines, these dimers were modeled using distance constraints between the residues corresponding to cysteine. Similarly, these three CALRs bind Ca^2+^ ions, stabilizing the structure, since the whole system is negative, contrary to the main CALRm classes A, B, and C (see Figure 6a,e,f). MDs were performed with similar parameters to before.

**Figure 10:**
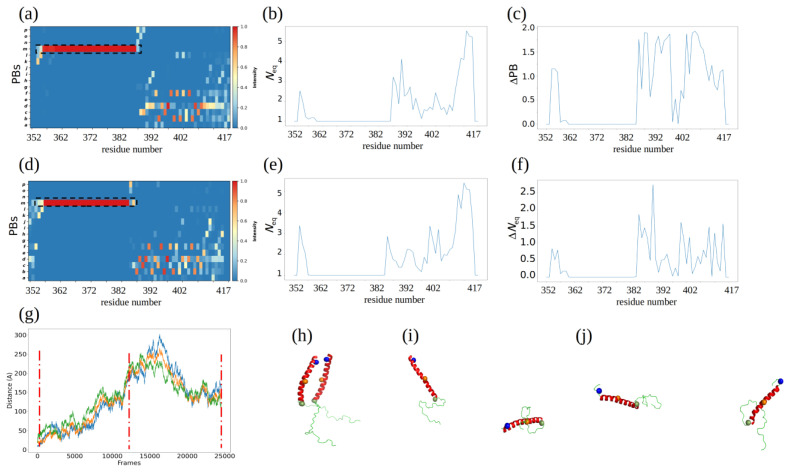
MD of CALRm dimer class E. PB analysis of (**a**) chain 1 and (**d**) chain 2; (**b**) and (**e**) show corresponding *N*_eq_; (**c**) ΔPB and (**f**) Δ*N*_eq_ between the two chains; (**g**) distance between different positions of the two chains (in blue, residue 356, in orange, 371, and in green, 386); and (**h**–**j**) 3D conformations observed during the simulations (positions in the simulation shown with red bars in (**g**)).

The dynamics of class E in the dimeric form was simulated over 250 nsec using Ca^2+^ ions to neutralize the system. As with the monomer, Ca^2+^ binding participates to stabilize each chain separately with some PB with high intensity (see Figure 10a,d). The helical regions remain well-structured and are more structured than the monomeric form of class E (see Figure 5e,f and Figure 10b,e). Indeed, the ΔPB between the chains and the monomer shows a value superior to one for the N-terminal part of the helix and, more specifically, for chain 1 (see Figure S11a), and it is slightly less for chain 2 (see Figure S11b). As with the other systems, the two chains have different flexibility properties (see Figure 10f and Figure S11b–d). As with the previous dimer, the distances between the chains were computed according to three anchor points (see Figure 10g). The chains do not remain in interaction and are quickly separated and reach a maximum distance of approximately 250 Å in 160 nsec. This is particularly striking when observing the different conformations observed in the simulation (see Figure 10h–j).

Another simulation was conducted using Na^+^ ions instead of Ca^2+^ ions (for a total duration of 110 nsec (see Video S14 and also Video S15 for comparison with CALRwt)). The helical regions remain stable with a slight unfolding of the helix C-cap. The second part of the sequence for each chain has a dynamic similar to the dynamics of CALRwt with Na^+^ ions. Indeed, the coil regions are free of binding since there is no Ca^2+^; therefore, they adopt a stable conformation, indicating that they are not moving a lot. For these regions, the ΔPB values between the chains and the CALRwt with Na^+^ ions are below one, which means that they share more similar dynamics in this particular region than seen in other systems (but remain highly dynamic).

The MD simulations of CALRm class D were conducted over 245 nsec using Ca^2+^ ions and 200 nsec using Na^+^ ions. The first simulation with Ca^2+^ ions shows a similar behavior of the ions as with the dimer of CALRwt. Ca^2+^ bindings stabilize the structure. Each chain has some variation in PB at the extremities of the helix, as with the monomeric form of class D with a ΔPB slightly above one. Some transient interactions could be provided by Ca^2+^ ions, punctually stabilizing the dimeric form, but these cases are not realistic due to the need for a high concentration of Ca^2+^ ions.

**Figure 11:**
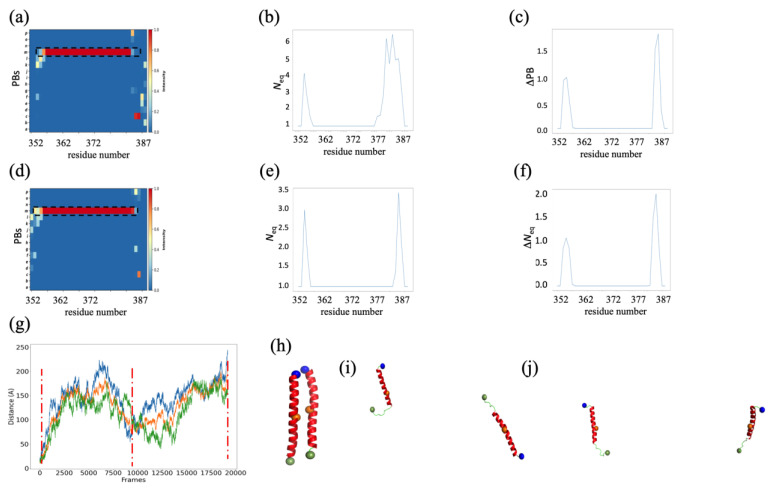
MD of CALRm dimer class D. PB analysis of (**a**) chain 1 and (**d**) chain 2; (**b**) and (**e**) show corresponding *N*_eq_; (**c**) ΔPB and (**f**) Δ*N*_eq_ between the two chains; (**g**) distance between different positions of the two chains (in blue, residue 352, in orange, 370, and in green, 388); and (**h**–**j**) 3D conformations observed during the simulations (positions in the simulation shown with red bars in (**g**)).

The simulation with Na^+^ ions shows a different dynamic (see Video S16). The helices are stable for both chains (see Figure 11a,d). However, the extremities of the helices are much more unstructured. The C-cap helix of chain 1 has an *N*_eq_ value of 6, which means that these regions are close to being considered as disordered regions, and a value of 3.5 for chain 2, i.e., still highly flexible (see Figure 11b,d). The ΔPB values range from 1.20 to 1.75, respectively (see Figure 11c), which is comparable with the values achieved with the monomeric class D (see Figure S12a–d). Hence, its extremities are flexible and sometimes close to being disordered regions (see Figure 11e). The distance calculation shows a clear separation between the chains, reaching an impressive distance of approximately 200 Å (see Figure 11g) and underlying the independent dynamics of both chains (see Figure 11i–k).

Thus, the results of this last part show that neither the CALRwt nor class E and class D are able to naturally form a dimer. They all keep the rigid/flexible dichotomy between the helical and non-helical regions, as seen with the different monomers (see Section 3.2). Hence, the absence of cysteines prevents a strong interaction between chains in order to remain stable. The different visualizations of the chains (see Figure 7i,j, Figure 10i,j, and Figure 11i,j) and corresponding distances (see Figure 7g, Figure 10g, and Figure 11g) show without any doubt that the chains move independently. Hence, these results clearly show that the C-domains of these two types of CALRm are strongly equivalent or even similar to those of CALRwt.

## 4. Discussion

Essential thrombocythemia is a blood cancer characterized by an overproduction of platelets mediated via a pathway in megakaryocytes involving variants of JAK2, MPL, and CALR [36,39,81]. Variants of CALR were classified into 3 groups: type 1, type 2, and other [82]. We thought that this classification could be improved to clearly cluster the properties of all CALRms. We previously proposed a novel classification based on five classes named A to E; this classification is different from the previous classification that underlines three types mainly centered on type I and II, which represent 80% of patients. The aim of our novel classification was to provide a better understanding of the role of CALRm in essential thrombocythemia cancer [44]. CALRm dimerization can lead to the activation of MPL via binding, thus inducing ET [36]. They are able to dimerize due to novel cysteines in their C-terminal sequences that come from frameshift mutations [52,83].

Our goal was to elucidate the dynamic properties of each class of CALRm and to investigate the viability of the dimerization of CALRms through disulfide bonds. Additionally, we wanted to verify our hypothesis that class E is only a polymorphism of CALRwt and is not associated with ET. To achieve this, we used molecular dynamics simulations to reproduce the theoretical dynamics of each CALRm class in monomeric and dimeric forms, and we analyzed them with Protein Blocks [71,84].

We, therefore, proposed a comparative modeling protocol to generate the 169 structural models of CALRms present to date in CALR-ETdb [47]. For this purpose, we used the C-domain from the PDB, which was incomplete and needed to be completed (please notice that the one obtained via the cryo-EM structure in [35] is the same as the one obtained previously via X-ray structures in [57,85], probably because of the molecular replacement). Predictions of secondary structures were made with PSI-PRED [76,86]; their confidence indices are excellent. In the first step, CALRwt and CALRm types I and II were built using MODELLER [58]. The other CALRms were derived from these, sometimes with other constraints to avoid having too-long helices. We mainly focused on CALRwt, CALRm class A (we took the classical type I as the reference for this), and CALRm class B (we took the classical type II as the reference for this class). CALRm class D corresponds well to the X-ray structure because it is very short. CALRm class E is simply CALRwt with a point mutation obtained using SCWRL [78]. For CALRm class C, we used class B as a support (see the alignment in Figure 1).

With the increase in Protein Data Bank structural data [87] and new Deep Learning approaches, we wanted to test (or re-test) these approaches. We made a wide choice with threading approaches: Phyre2 [64], de novo, i.e., I-TASSER [61], and Robetta [62], and Deep Learning AlphaFold2 [63], locally, and also with CollabFold (on a reduced basis, as in [88]). The confidence indices remain very low for the disordered part of CALRwt (similar to class E) but also for classes A, B, and C. We have to remember that the new sequences obtained via the frameshifts do not exist in nature, and are not, therefore, in the databases, i.e., the multiple sequence alignments are completely empty and cannot provide any information. Therefore, we kept our models (see Data S1).

From a dynamic point of view, it is interesting to see that in the ER, the Ca^2+^ ions allow CALRwt and the two CALR classes D and E to strongly rigidify them. The number of possible Ca^2+^ binding sites is impressive and made such that depending on the simulation, they could be different. These results are in good agreement with the previous experimental and predicted results [37,38,52,83,89]. Indeed, these results show that disulfide points are of major importance for the dimerization of the C-domain of CALRm. Venkatesan and co-workers effectively highlighted this with type I, our class A. We reproduced these results by mutating the cysteines, reaching the same conclusion [52,53,83]. The analysis of the dynamics is also in good agreement with analyses performed using predictive approaches for disorders by Uversky and collaborators [37,89]. They highlighted the difficulty of seeing ordered areas in C-termini [38]. Herein, we presented, in addition, the localization of rigid fragments surrounded by very deformable regions (*N*_eq_ > 6). This leads to the localization of mobile zones [74] usable for therapeutic research approaches.

In more detail, we also highlighted the possibility of having strongly constrained areas in disordered regions (see Figure 3). The three classes with frameshifts and losses of the retention signal also have strongly disordered regions (see Figure 4) but show very different behaviors. The cases of CALRm class A (canonical type I) and CALRm class B (canonical type II) are striking, such that the first one has a particularly flexible C-terminal region, while for the second one, three helices are observed with an intermediate disordered region (thus, in agreement with the previous analyses [37,38,89] but more precise in terms of the structural characteristics).

The analysis of dimers is more complex because the flexible parts are more difficult to analyze. However, CALRm classes A, B, and C necessarily need the creation of the disulfide bridges to remain associated. CALRwt and CALRm classes D and E show no affinity for remaining in dimer form. The examples of broken disulfide bonds in CALRm class A highlight the necessity of having disulfide bonds in these variants and, more importantly, indicate that these cysteines are probably determinants of ET. An interesting and very reassuring point is that it is not necessary to carry out very long dynamics to arrive at a sufficient conformation sampling. Indeed, with less than 200 nsec of simulations, all the conformations were observed, and the values of RMSF and *N*_eq_ no longer evolved. We have carried out tests over much longer times with several replicas (the data are not shown).

To conclude, Table 1 summarizes most of our findings. Our results suggest that class E is effectively very similar to CALRwt in terms of its dynamics and behavior in monomeric and dimeric forms. Hence, our hypothesis that class E is not associated with ET could be correct, and it may only correspond to human polymorphism. Class D is roughly half of CALRwt and has different dynamics compared with CALRwt but also cannot dimerize due to the absence of cysteines in its sequence, which is similar to CALRwt and class E. It may also not be a driving force of ET. We hope that these results will lead to new reflections on the sequencing of ETs as well as on therapeutic possibilities.

These results are important as ET is associated with a large number of complications [9], and it remains difficult to associate the clinical significance with the prognoses of the different CALR types [90,91]. Herein, we underlined that these types must also be carefully taken into consideration. These results are also critical in regard to the search for new drugs and antibodies focusing on CALRms [40,92].

## Figures and Tables

**Table 1:** Provided for each system are the length of CALR fragment, the presence (or not) of ER retention signal, the complete charge of the fragment, the % of residues associated with helical content, the presence (or not) of cysteine, and whether the dimer is stable.

System	Length	KDEL	Charge	Helix (%)	Cysteine	Stable Dimer
CALRwt	66	Yes	26 −	43	No	No
CALRm A	60	No	15 +	38	Yes	Yes
CALRm B	79	No	9 +	55	Yes	Yes
CALRm C	78	No	15 +	28	Yes	Yes
CALRm D	37	No	9 −	89	No	No
CALRm E	66	Yes	26 −	43	No	No

## Data Availability

Not applicable.

## Supplementary Materials

The following supporting information can be downloaded at https://www.mdpi.com/article/10.3390/biom13030509/s1. Figure S1: Ca^2+^ binding with CALRwt. Figure S2: CALRwt MD (100 nsec). Figure S3: CALRwt MD, an example of Ca^2+^ impact on the local conformations. Figure S4: MD simulations of CALRwt with Ca^2+^, Na^+^, and Cl^-^. Figure S5: CALRm class E. Figure S6: dimers of CALRm classes A, B, and C. Figure S7: comparison of CALRwt chains of the dimer and monomer alone. Figure S8: comparison of CALRm class A chains of the dimer and monomer alone. Figure S9: dimers of CALRm class A without disulfide bridge. Figure S10: comparison of CALRm class B chains of the dimer and monomer alone. Figure S11: comparison of CALRm class E chains of the dimer and monomer alone. Figure S12: comparison of CALRm class D chains of the dimer and monomer alone. Video S1: CALRwt molecular simulation with Ca^2+^ ions binding to the structure. Video S2: CALRwt molecular simulation with calcium ions binding to the structure. Video S3: CALRwt molecular simulation with Na^+^ ions. Video S4: CALRm class A molecular simulation. Video S5: CALRm class B molecular simulation. Video S6: CALRm class C molecular simulation. Video S7: CALRm class D molecular simulation. Video S8: CALRm class E molecular simulation. Video S9: dimeric form of CALRm class A molecular simulation. Video S10: dimeric form of CALRm class A with broken disulfide bonds molecular simulation. Video S11: dimeric form of CALRm class B molecular simulation. Video S12: dimeric form of CALRm class B molecular simulation. Video S13: dimeric form of CALRm class C molecular simulation. Video S14: dimeric form of CALRwt molecular simulation. Video S15: dimeric form of CALRm class E molecular simulation. Video S16: dimeric form of CALRm class D molecular simulation. Data S1. All the initial structural models for the monomers and dimers used in this study.
