# Supplementary material for: Structural and Dynamic Differences between Calreticulin Mutants Associated with Essential Thrombocythemia
Source: Biomolecules. 2023 Mar 10;13(3):509. doi: 10.3390/biom13030509 (PMC10046389; doi:10.3390/biom13030509)
Supplement: Supplementary file 1 [file biomolecules-13-00509-s001.zip › biomolecules-2155679-supplementary.pdf]

**Title:** Structural and dynamic differences between calreticulin mutants associated with essential thrombocythemia.

**Authors:** Ragousandirane Radjasandirane & Alexandre G. de Brevern*

(a) (b)

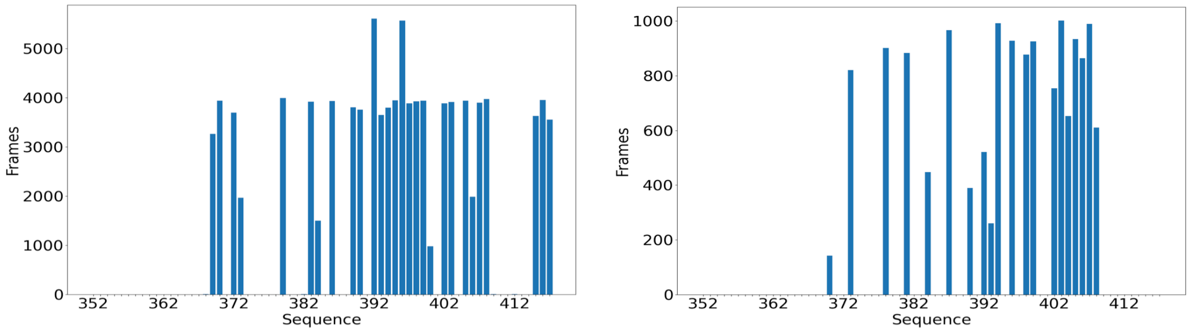


**Figure S1**. *$Ca^{2+}$ binding with CALRwt*. MD simulations of (a) 400 nsec and (b) 100 nsec.

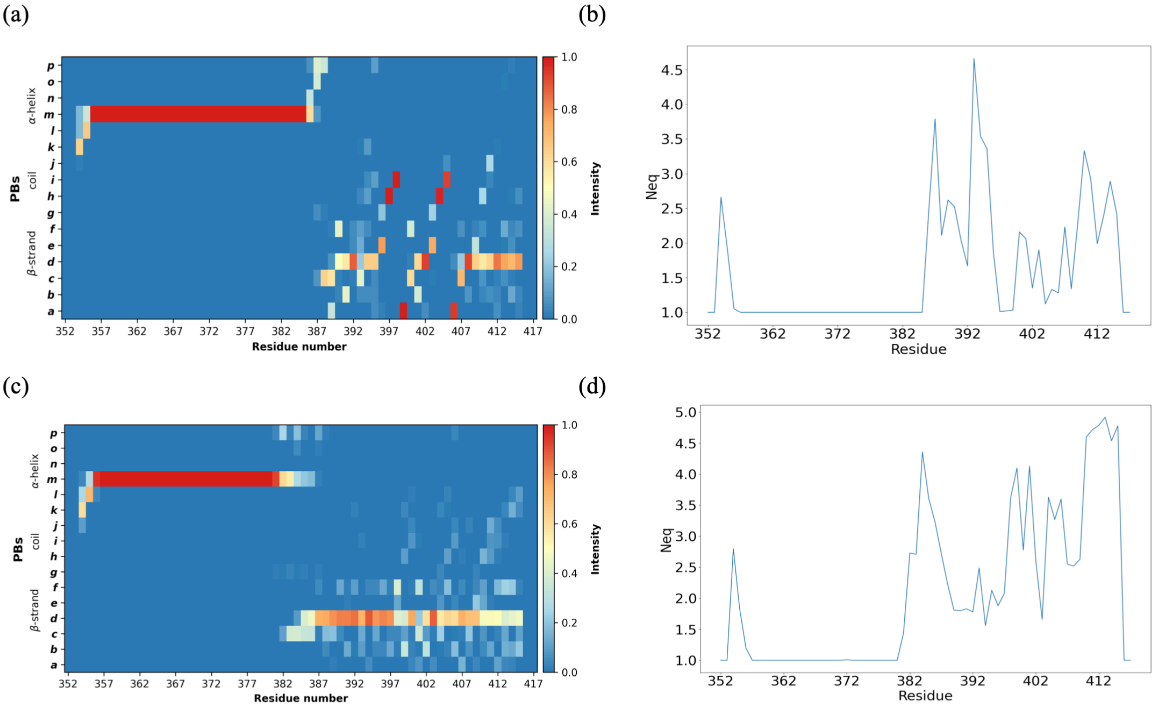


**Figure S2**. *CALRwt MD (100 nsec)*. (a) and (c) PB analysis, and (b) and (d) corresponding $N_{eq}$ for (a) and (b) $Ca^{2+}$ and (c) and (d) $Na^{+}$.

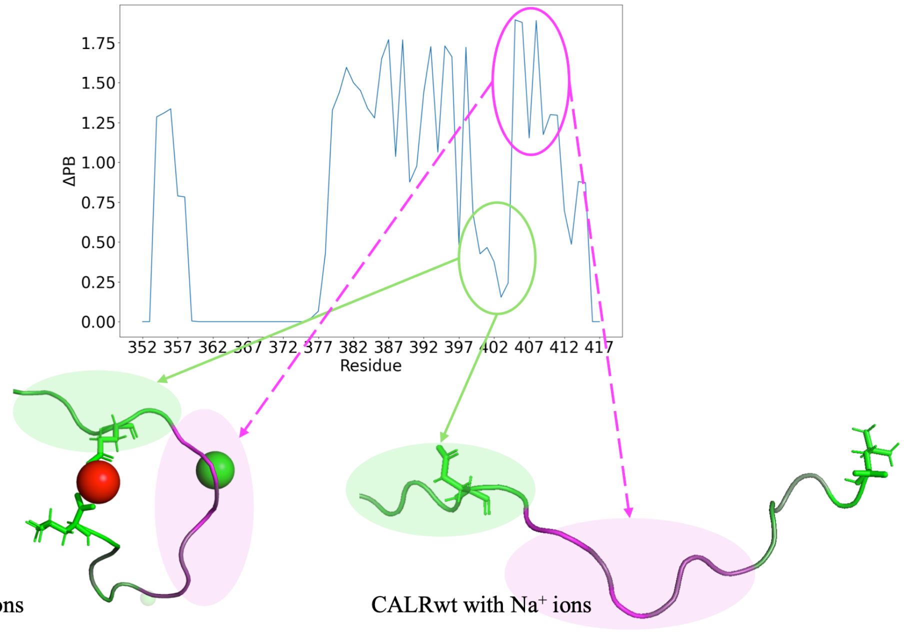


CALRwt with $Ca^{2+}$ ions

CALRwt with $Na^{+}$ ions

**Figure S3**. *CALRwt MD: an example of $Ca^{2+}$ impact on the local conformations*. Is underlined the importance of $Ca^{2+}$ binding between E403 and L417.

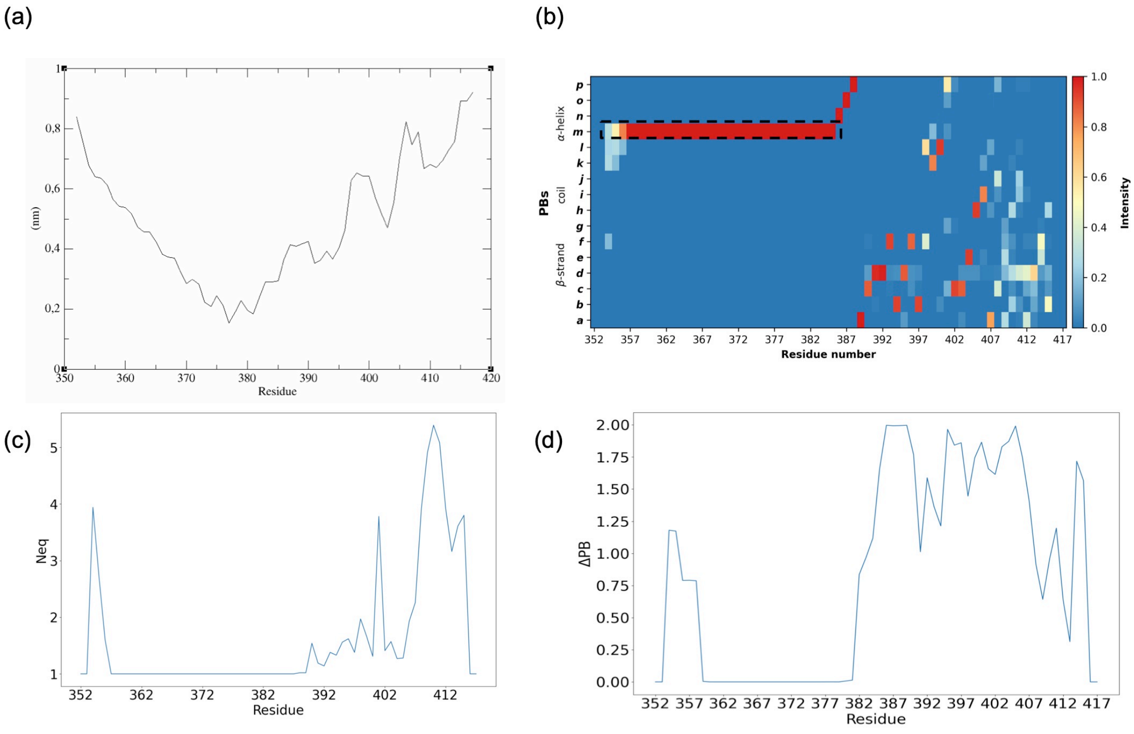


**Figure S4**. *MD simulations of CALRwt with $Ca^{2+}$, $Na^{+}$ and $Cl^{-}$*. (a) RMSF, (b) PB analysis, (c) $N_{eq}$ and (d) ΔPB with MD of CALRwt only with $Ca^{2+}$.

The dynamic characteristics of the CALRwt simulation with $Ca^{2+}$, $Na^{+}$ and $Cl^{-}$ ions are fully comparable to that of CALRwt with Ca2+ ions only. The helix has the same length and remains stable all the time (see Figures 3a, 3c, S4a, S4b and S4c). The $Ca^{2+}$ sites are very quickly used, and as seen in the other replicates, they are not exactly the same all the time (see Figures S2a and S2b), this is found here and thus gives a large ΔPB in the charged C-termini region (see Figure S4d).

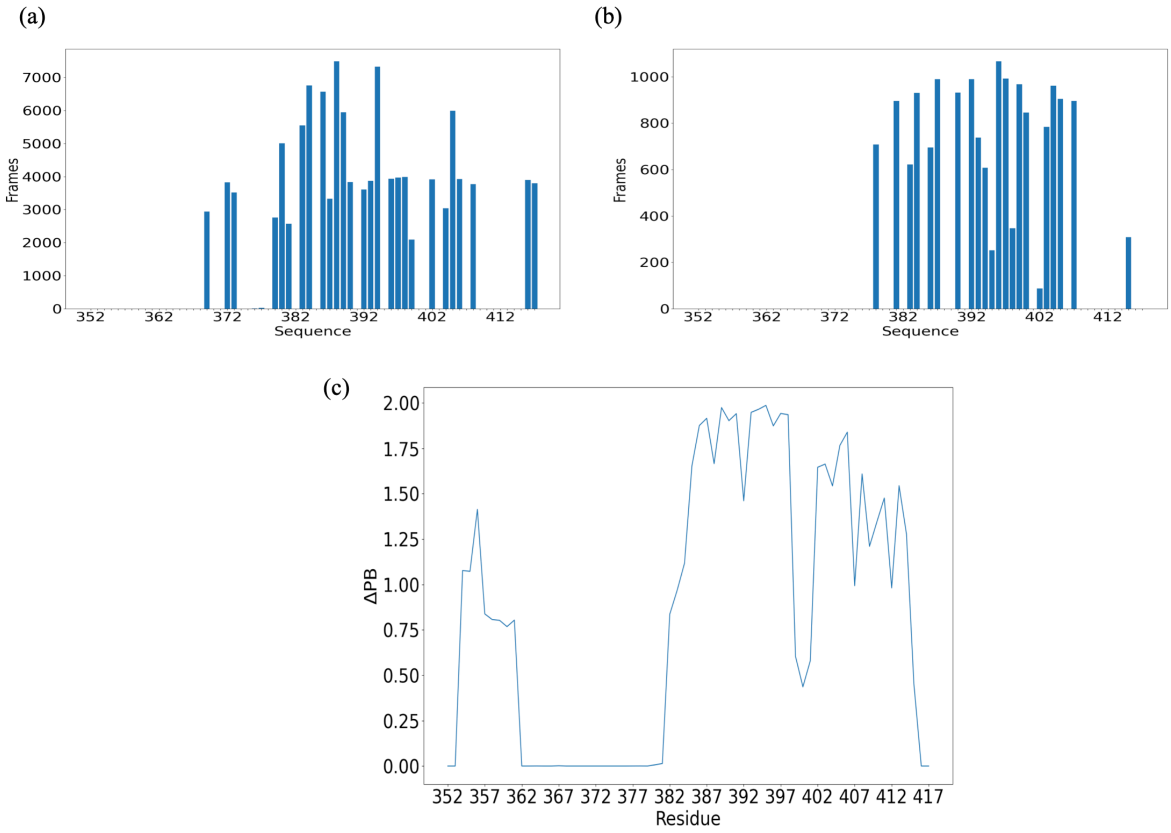


**Figure S5**. *CALRm class E*. $Ca^{2+}$ binding for MD simulations of (a) 400 nsec and (b) 100 nsec; (c) ΔPB of CALRwt and CALRm class E.

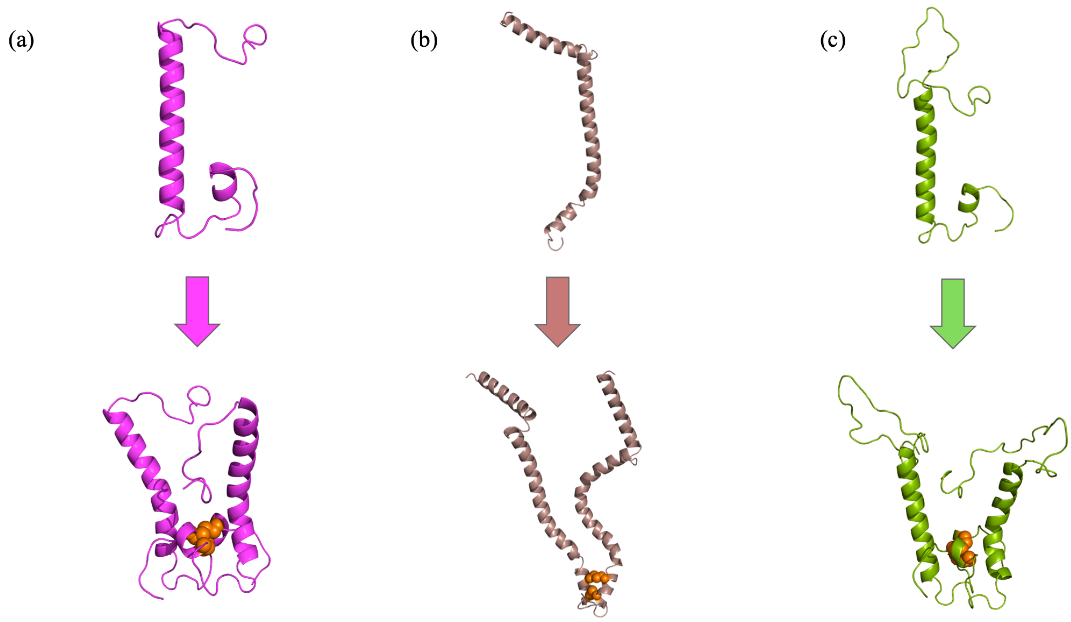


**Figure S6**. *Dimers of CALRm classes A, B and C*. From a monomer, the dimer is build to allow a correct disulphide bridge made by Cysteines for (a) CALRm A, (b) CALRm B and (c) CALRm C.

CALRm class A, B and C can activate the proliferation process of platelets by binding MPL surface receptors of megakaryocytes through a dimeric form. Indeed, these variants are affected by a frameshift mutation that produces a new sequence in which the KDEL motif is absent; therefore, they are no longer maintained in the ER. These new sequences present two cysteines that allow a dimeric form of these CALRm through disulphide bonds between monomers. The dimeric form of these 3 classes is modelled using MODELLER and the crystal structure PDB id 5LK5 (it is identical to PDB id 6ENY chain E structure [1]) as template, which contains a 10-mer of CALR D71K mutant. We simulated the dynamics of each class in their dimeric form to further characterize the impact of the disulphide bonds newly formed on the dynamics and stability of the structure.

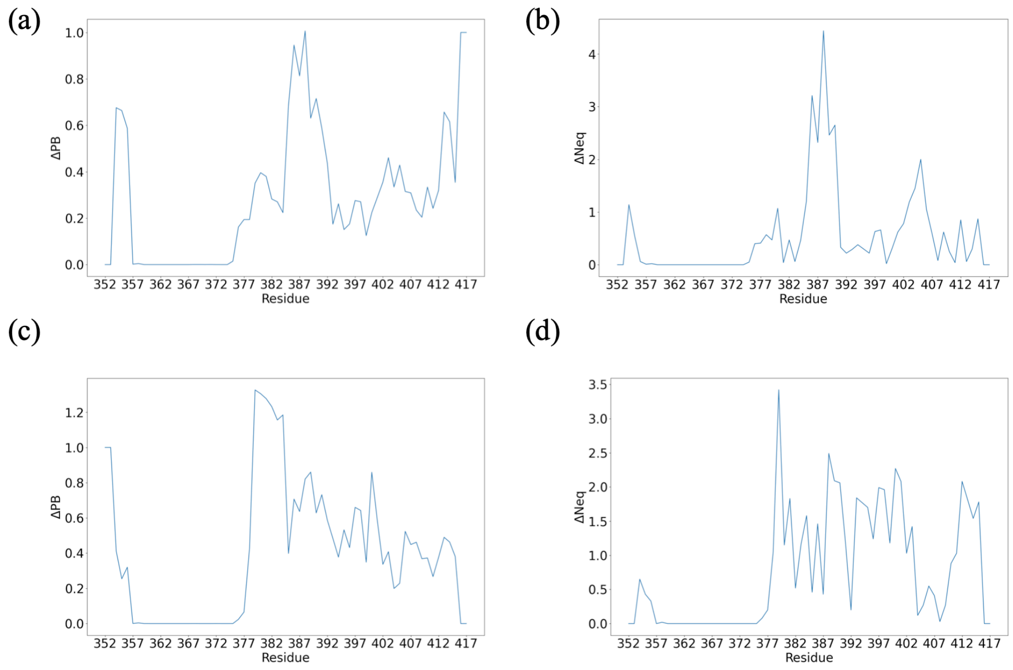


**Figure S7**. *Comparison of CALRwt chains of the dimer and monomer alone*. (a) and (c) ΔPB of chains 1 and 2 of CALRwt dimer *vs* CALRwt monomer, (b) and (d) corresponding $\Delta N_{eq}$.

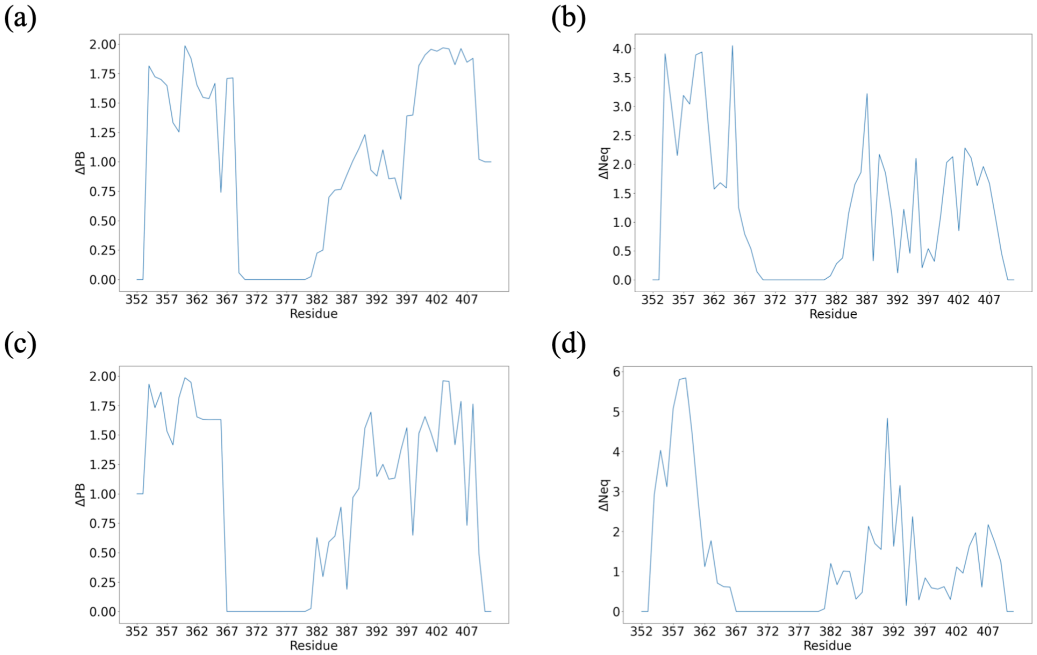


**Figure S8**. *Comparison of CALRm class A chains of the dimer and monomer alone*. (a) and (c) ΔPB of chains 1 and 2 of class A dimer *vs* class A monomer, (b) and (d) corresponding $\Delta N_{eq}$.

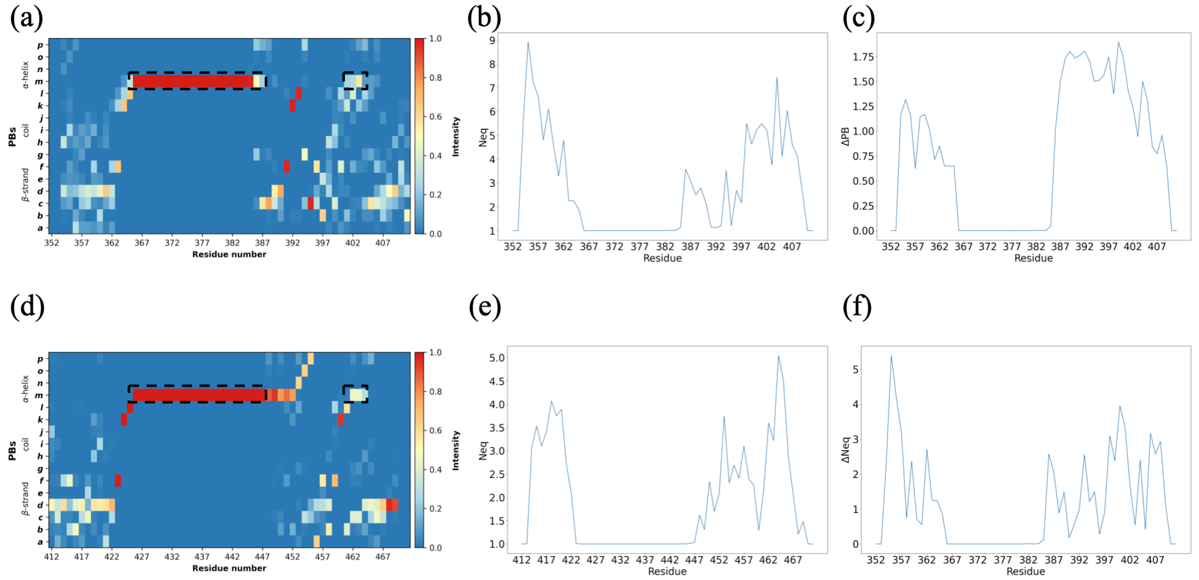


**Figure S9**. *Dimers of CALRm class A without disulphide bridge*. PB analysis of (a) chain 1 and (d) chain 2, (b) and (e) corresponding $N_{eq}$, (c) ΔPB and (f) $\Delta N_{eq}$.

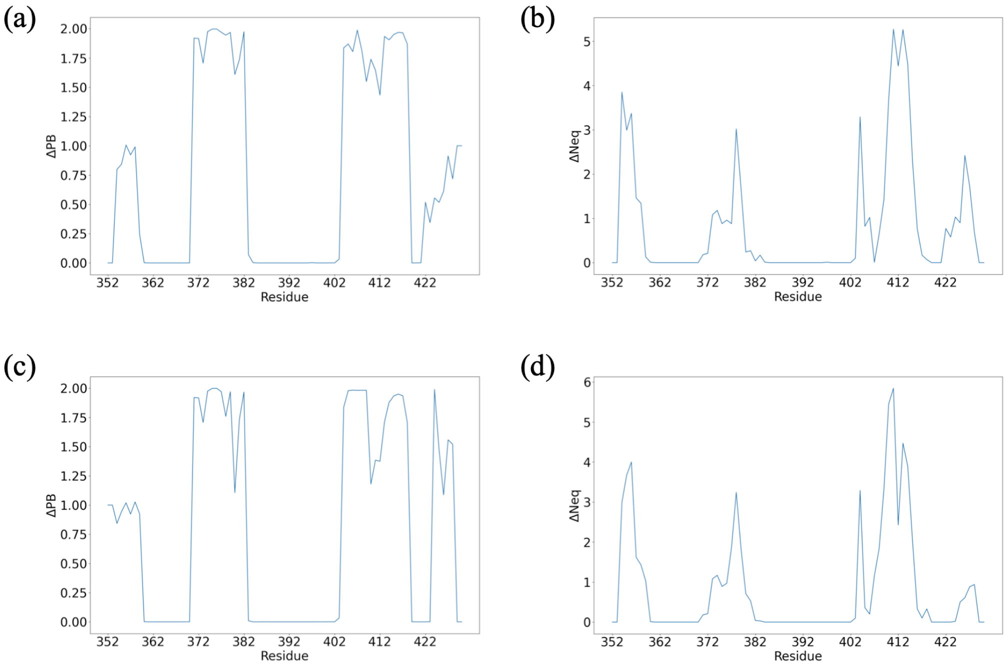


**Figure S10**. *Comparison of CALRm class B chains of the dimer and monomer alone*. (a) and (c) ΔPB of chain 1 and 2 of class B dimer *vs* class B monomer, (b) and (d) corresponding $\Delta N_{\mathrm{eq}}$.

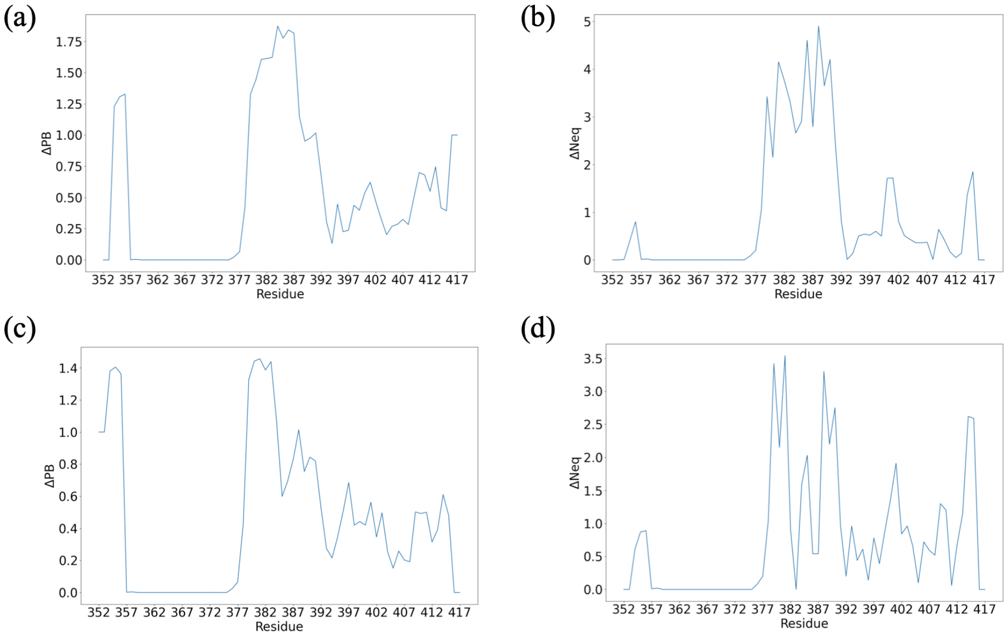


**Figure S11**. *Comparison of CALRm class E chains of the dimer and monomer alone*. (a) and (c) ΔPB of chain 1 and 2 of class E dimer *vs* class E monomer, (b) and (d) corresponding $\Delta N_{eq}$.

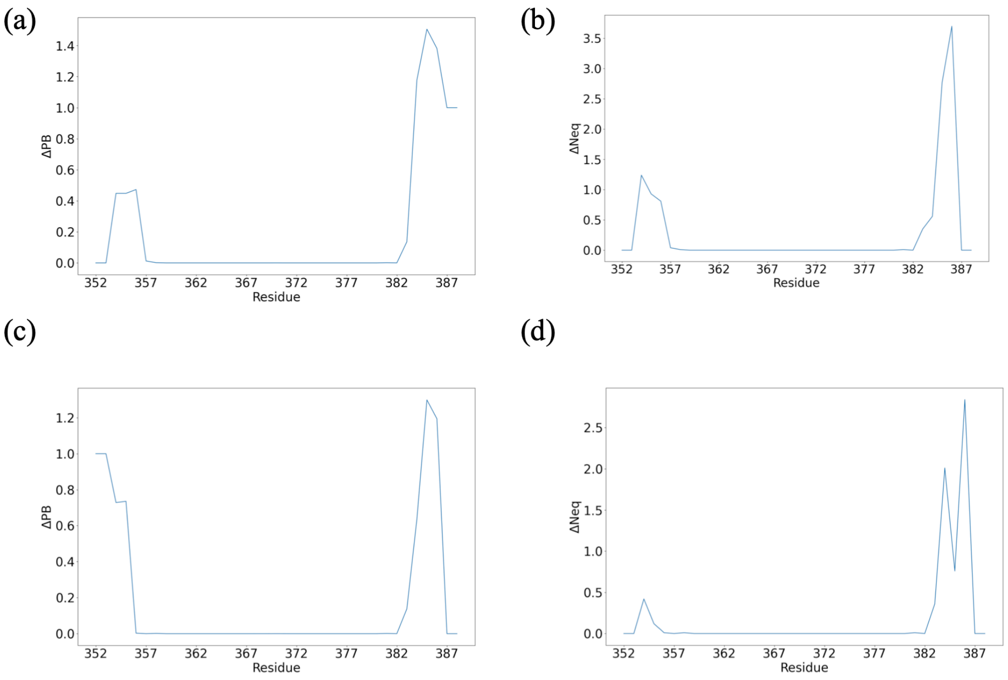


**Figure S12**. *Comparison of CALRm class D chains of the dimer and monomer alone*. (a) and (c) ΔPB of chain 1 and 2 of class D dimer *vs* class D monomer, (b) and (d) corresponding $\Delta N_{eq}$.

**Supplementary Videos** (can found at https://zenodo.org/record/7486489#.Y6rgauzMJTY and https://www.dsimb.inserm.fr/~debrevern/ET/Videos/)

**Video S1**. *CALRwt molecular simulation with $Ca^{2+}$ ions binding to the structure.* This movie shows the dynamics of $Ca^{2+}$ ions over the 40 ns and how they rapidly bind to the CALRwt structure. Most of the ions bind to the C-terminal part of CALRwt, where the majority of the negative residues are located. The binding of $Ca^{2+}$ ions creates an interaction between residues that can induce specific folding. In this example, residues E407 and E416 interact with a calcium ion and fold, as shown at the end of the movie.

**Video S2.** *CALRwt molecular simulation with calcium ions binding to the structure.* This movie shows the dynamics of CALRwt and $Ca^{2+}$ ions over 400 ns and how ions are rapidly bound to the CALRwt structure. Most of the ions bind to the C-terminal region of CALRwt, where the majority of the negative residues are located. The binding of calcium ions creates an interaction between residues that can induce specific folding (a specific example of fold is shown on Video S1). On this full movie, we are able to see the unfolding of the N-terminal region of the helix and the stabilization of the C-terminal region thanks to calcium binding.

**Video S3**. *CALRwt molecular simulation with $Na^{+}$ ions.* This movie shows the dynamics of CALRwt with $Na^{+}$ ions. The absence of binding from calcium ions leaves the structure free of any external constraint, especially for the C-terminal region. The latter appears to be flexible but this region is in fact quite stable at the local level and has its own dynamics, unstructured slightly the C-terminal of the helix.

**Video S4**. *CALRm class A molecular simulation.* This movie shows the dynamics of CALRm class A over 400ns. The C-terminal region of the structure is quite flexible at first and interacts with the N-terminal region after several ns. This interaction locally constrains the structure around residues 400-404, stabilizing an unstructured structure for this region.

**Video S5**. *CALRm class B molecular simulation.* This movie shows the dynamics of CALRm class B over 400ns. The first and last helices move away from their initial position with great flexibility of the coiled regions between the helices. The first helix interacts closely with the second helix and forms a specific T-shaped fold which stabilize the whole structure.

**Video S6**. *CALRm class C molecular simulation.* This movie shows the dynamics of CALRm class C over 400ns. Extremities are highly flexible but the helix remains stable. This high flexibility allow the C-terminal region to have some interaction with the helix at some frames

**Video S7**. *CALRm class D molecular simulation.* This movie shows the dynamics of CALRm class D over 400ns. The C-terminal region appears to be flexible, but the helix remains stable the whole simulation.

**Video S8**. *CALRm class E molecular simulation.* This movie shows the dynamics of CALRm class E over 400ns. This movie shows the dynamics of CALRm class E and calcium ions over 400 ns and how ions are rapidly bound to the CALRwt structure. Most of the ions bind to the C-terminal region of *CALRm class E,* where the majority of the negative residues are located. The N-terminal region of the helix is being unstructured and the C-terminal region is stabilized with calcium ions. This simulation of CALRm class E is very similar to the simulation of CALRwt.

**Video S9.** *Dimeric form of CALRm class A molecular simulation*. This movie shows the dynamics of two CALRm class A monomers forming a dimer through disulphide bonds. Both chains seem to repulse each other due to electrostatic charges, but disulphide bonds maintain the dimeric form, otherwise both chains would have been separated.

**Video S10.** *Dimeric form of CALRm class A with broken disulphide bonds molecular simulation*. This movie shows the dynamics of two CALRm class A monomers and their attempt to form a dimer with broken disulphide bonds. As each monomer repels each other, the dimeric form cannot be stable without any disulphide bonds. This is demonstrated by the separation of each chain from each other.

**Video S11.** *Dimeric form of CALRm class B molecular simulation*. This movie shows the dynamics of two CALRm class B monomers forming a dimer through disulphide bonds. Both chains are interacting together to form a specific shape, similar to the simulation of the monomer of class B. This interaction implies that even without any disulphide bonds, the dimeric form of class B could be stable, contrary to class A.

**Video S12.** *Dimeric form of CALRm class B molecular simulation*. It is an interesting replicate of the same system than Video S11. Helices are also well maintained.

**Video S13.** *Dimeric form of CALRm class C molecular simulation*. This movie shows the dynamics of two CALRm class C monomers forming a dimer through disulphide bonds. Both chains seem to repulse each other due to electrostatic charges, but disulphide bonds maintain the dimeric form, otherwise both chains would have been separated.

**Video S14.** *Dimeric form of CALRm class E molecular simulation.* This movie shows the dynamics of two CALRm class E monomers and their attempt to form a dimer without any disulphide bonds. Chains repel and are moving away from each other after several ns, indicating the inability for class E to form a dimer.

**Video S15.** *Dimeric form of CALRwt molecular simulation*. This movie shows the dynamics of two CALRwt monomers and their attempt to form a dimer. Some interactions occur between the N-terminus of each chain, but this is not sufficient and the chains move away from each other. Subsequently, the dynamics of each chain resembles the dynamics of the CALRwt monomer simulated with sodium ions (Video S2). CALRwt is not able to be stable as dimer.

**Video S16.** *Dimeric form of CALRm class D molecular simulation.* This movie shows the dynamics of two CALRm class D monomers and their attempt to form a dimer without any disulphide bonds. Both chains are separated very quickly which indicate that they cannot be stable as dimer.

**References**


1. Blees, A.; Januliene, D.; Hofmann, T.; Koller, N.; Schmidt, C.; Trowitzsch, S.; Moeller, A.; Tampé, R. Structure of the human mhc-i peptide-loading complex. *Nature* **2017**, *551*, 525-528.
